# Multipath Projection Stereolithography for Three-Dimensional Printing Microfluidic Devices

**DOI:** 10.1021/acsami.4c10547

**Published:** 2024-12-03

**Authors:** Zachary J. Geffert, Zheng Xiong, Jenna Grutzmacher, Maximilian Wilderman, Ali Mohammadi, Alex Filip, Zhen Li, Pranav Soman

**Affiliations:** 1 Department of Biomedical and Chemical Engineering, Syracuse University, 900 S Crouse Avenue, Syracuse, New York 13244, United States; 2 Department of Mechanical Engineering, Clemson University, 105 Sikes Hall, Clemson, South Carolina 29634, United States; 3 3D Microfluidics LLC, 5900 Strawmount Trail, Chittenango, New York 13037, United States

**Keywords:** multiscale, 3D printing, precision microfluidics, photopolymerization, additive manufacturing

## Abstract

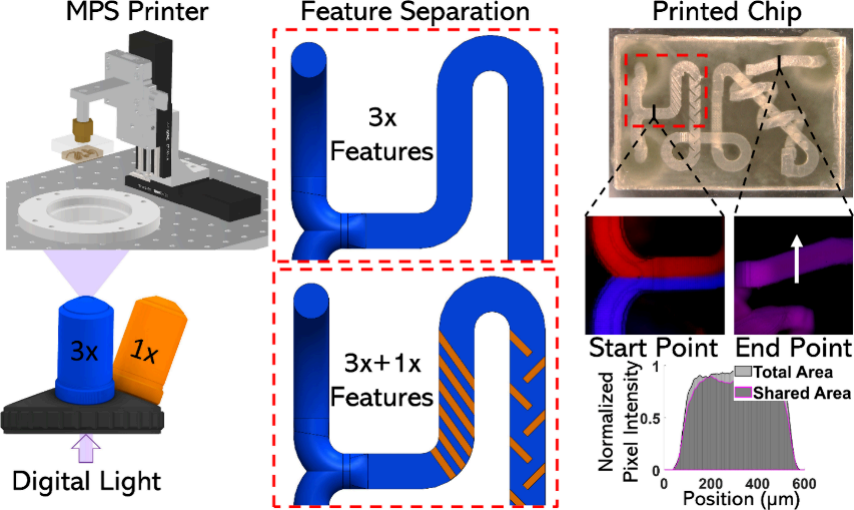

Although many lab-on-chip applications require inch-sized devices with microscale feature resolution, achieving this via current 3D printing methods remains challenging due to inherent trade-offs between print resolution, design complexity, and build sizes. Inspired by microscopes that can switch objectives to achieve multiscale imaging, we report a new optical printer coined multipath projection stereolithography (MPS) specifically designed for printing microfluidic devices. MPS is designed to switch between high-resolution (1× mode, ∼10 μm) and low-resolution (3× mode, ∼30 μm) optical paths to generate centimeter-sized constructs (3 × 6 cm) with a feature resolution of ∼10 μm. Illumination and projection systems were designed, resin formulations were optimized, and slicing software was integrated with hardware with the goal of ease of use. Using a test case of micromixers, we show that user-defined CAD models can be directly input to an automated slicing software to define printing of low-resolution features via the 3× mode with embedded microscale fins via 1× mode. A new computational model, validated using experimental results, was used to simulate various fin designs, and experiments were conducted to verify simulated mixing efficiencies. New 3D out-of-plane micromixer designs were simulated and tested. To show broad applications of MPS, multichambered chips and microfluidic devices with microtraps were also printed. Overall, MPS can be a new fabrication tool to rapidly print a range of lab-on-chip applications.

## 1 Introduction

Microfluidic devices that enable the control and manipulation of microliter volumes of liquid are widely used for many applications. Photolithography remains the gold standard to make such devices; however, due to the need for cleanroom and microfabrication facilities with technical expertise, specialized equipment, and labor-intensive steps (plasma bonding, PDMS molding, device assembly), this remains time- and cost-prohibitive, especially for low production volume or high complexity designs. As an alternative to cleanrooms, 3D printing methods such as fused deposition modeling (FDM) and vat photopolymerization (VPP) methods have been used to print microfluidic devices. However, due to the low feature resolution of FDM (∼100 μm), VPP methods have emerged as the method of choice to print high-resolution microfluidic devices. VPP relies on light irradiation from a laser spot (vector scanning) or digital mask projections (DLP) to initiate polymerization and print 3D objects in a layer-by-layer manner. Since the vector scanning approach is limited by long scanning times and complex process planning systems, DLP-VPP has emerged as the leading method for making microfluidic devices. In this method, a UV light source is spatially modulated by a digital micromirror device (DMD) to generate pixelated light patterns derived from a sliced CAD model. DLP-VPP has been widely adopted to print miniaturized chips using custom and commercial printers and resins with channel sizes ranging from 25 to 150 μm.^1−10^ However, since the pixel number is based on the number of micromirrors on a DMD chip (1920 × 1080), the projection area is inversely proportional to the feature resolution. For instance, a build area of 48 mm × 36 mm^11^ would have a resolution of ∼50 μm while 1 μm resolution can be only achieved by scaling down to a print area of only 2 mm × 1 mm, an impractical size for most lab-on-chip applications.^12^ To enable easy adoption by researchers, printed devices should fit onto a standard microscope slide (75 mm × 26 mm); however, this would require multiscale DLP-VPP strategies, as discussed below.^7,13−19^

The most common type is the use of the motorized step-stitching method that involves dividing the CAD file into a series of steps, moving either a motorized stage or the digital light engine by a defined distance before irradiation of pixelated images.^20,21^ Here, since the print area per exposure does not change, feature resolution remains high. However, key limitations including stitching errors between adjacent regions despite sophisticated image processing methods and longer fabrication times due to multiple stage movement and exposure steps remain. To reduce print times, concurrent light projection and stage movement have also been developed,^22−24^ however high image refresh rate to avoid motion blurring during printing requires custom graphics hardware, which has limited its utility in the field. The strategy of mounting multiple projectors to cover a larger area involves high costs and alignment issues. Another strategy is to use two distinct light sources, one to print low-resolution and typically internal features and second, a high-resolution laser to print contours.^25−27^ To maintain high print speeds and resolution, pixel blending methods have been developed however this remains computationally prohibitive.^28^ Hybrid machines have also been built that integrate laser scanning using galvo mirrors with DLP-VPP; however, high cost, complex process planning, and low print speed and spot positioning errors during laser scanning remain a challenge.^29^ Recently, two-axis galvo mirrors combined with a custom f-theta lens and novel hopping light DLP offer promising solutions for multiscale printing.^30^ Combining vector scanning and DLP-VPP involves complex process controls to coordinate the slicing algorithm with laser path planning and mask generation.^25,31−33^ Machines with integrated vertical and rotatory degrees of freedom have also been used for large-area printing; however, complex control systems have limited their utility in the field.^34^ Overall, the complexity of such multiscale DLP platforms prevents their adoption within nonspecified broader communities.

With ease of use as our inspiration and microfluidic chips as the target application, we set out to design a printer that could print devices that would fit onto a standard microscope slide (75 mm × 26 mm) yet maintain a feature resolution of ∼10 μm without significantly increasing process complexity, printing time, or hardware costs. Here, we report a new multipath projection stereolithography (MPS) printer capable of rapid multiscale printing of parts as large as 30 mm × 60 mm (1.8 × 2.36 in.) sized microfluidic devices and structures with ∼10 μm resolution. MPS consists of a single light source and two optical configurations that can be switched between 3× mode (resolution of ∼30 μm) and 1× mode (resolution of ∼10 μm) to realize multiscale microfluidic devices. Both lateral and vertical resolutions for each mode were characterized. The ability to print 3D structures with complex designs was demonstrated by using an Empire State Building and alveolar model with complex internal fluidic topologies. Using micromixers as a test case, we show that MPS can rapidly design and print devices with variations in fin type based on target mixing efficiency derived from fluid flow simulations. We also show the printing and testing of micromixers with complex 3D out-of-plane channel topologies. Lastly, we report that MPS can be used to rapidly design and print microfluidic devices that cannot be printed by 1× or 3× mode used in isolation; here, the 3× mode was used to print macroscale features, while smaller microscale features were printed using 1× mode.

## 2 Materials and Methods

### 2.1 PEGDA Prepolymer Preparation

Poly(ethylene glycol) diacrylate (PEGDA, Mn = 250) and the photoinitiator, phenylbis (2,4,6-trimethylbenzoyl) phosphine oxide (Irgacure 819), were purchased from Sigma-Aldrich and used without any further modifications. The photoabsorber 2-Isopropylthioxanthone (ITX) was purchased from the Tokyo Chemical Industry and used without further modifications. The prepolymer solution was composed of PEGDA (100% v/v) with Irgacure 819 (1% w/v) and ITX (1.5% w/v). The prepolymer solution was mixed with a stainless-steel stirrer, then vortexed, and placed in a water bath at 37 °C repeatedly until Irgacure 819 and ITX had dissolved.

### 2.2 Fabrication

The material vat consists of a polystyrene Falcon brand 100 mm × 15 mm Petri dish with a poly(dimethylsiloxane) (PDMS) buffer cured to the bottom of the dish. Approximately 3.5 g of PDMS is poured into the Petri dish, vacuum degassed to remove entrained air bubbles, and heat cured at 60 °C overnight.

### 2.3 Methacrylation of Glass Coverslip

Glass coverslips were immersed into 10% (w/v) NaOH solution for 30 min and washed in DI water, 75% (v/v) ethanol, and 100% ethanol (performed twice for 3 min for each wash). The coverslip was subsequently dried by using nitrogen. The dried coverslips then underwent methacrylation by immersing them for 12 h in a solution comprised of 85 × 10^–3^ M 3-(trimethoxysilyl) propyl methacrylate (TMSPM, Sigma) and ethanol solution with acetic acid (pH 4.5). Finally, the coverslips were washed with ethanol three times and baked for 1 h at 100 °C.

### 2.4 Fluorescent Dyes

Two mg/mL of 150 kDa FITC-dextran and 1 mg/mL of 70 kDa rhodamine-dextran were mixed in a DI water solution.

### 2.5 SEM

For obtaining the SEM (JSM 5600, JEOL, Japan) images, samples were separated from their printing mount, washed with ethanol, and dried. Then, samples were sputter coated (Vacuum Desk V, Denton, Moorestown, NJ) for 45 s with a layer of gold and imaged under SEM with 10 kV accelerating voltage.

### 2.6 Micro-CT Analysis

Following printing, the microfluidic chips were washed with ethanol and placed on a solid 3D printed base with double-sided tape to prevent movement. The base was placed inside a 20 mm diameter sample holder for micro-CT imaging (micro-CT 40, Scanco Medical AG, Brüttisellen, Switzerland). Imaging was conducted at a 10 μm isotropic voxel resolution using 55 kV, 145 mA, and a 200 ms integration time. Following scanning, the reconstructed images (.isq files) were transferred into Materialize Mimics, a 3D medical image segmentation software, for analysis. Images were then cropped to isolate the microfluidic chips, and a global threshold of 200 mg HA cm^–3^ was applied. A 3D reconstruction was generated from these data and exported as an STL file for visualization purposes.

### 2.7 CFD

To reduce the cost and time for physical prototypes and accelerate the microfluidics development process, we employed computational fluid dynamics (CFD) simulations implemented in ANSYS Fluent to allow for rapid prototyping in a virtual environment. CFD studies provide us with detailed insights into flow patterns, mixing features, and concentration distributions in microfluidic channels with various microscale fins. This predictive capability helps in anticipating the behavior of the fluid flow and mixing before an actual microfluidic channel is fabricated. CFD simulations are used to optimize the design of microfluidic channels, including adjusting channel geometries and structures, flow rates, and other parameters, to achieve efficient mixing along the flow. Details of the CFD model and simulation results and corresponding analysis are included in the Supporting Information, Section 5.

### 2.8 Components/Devices for System Design

The optical and optomechanical components are purchased from Thorlabs, Edmund Optics, and RPC photonics. Other customized mechanical components such as a rotator for the engineered diffuser, polymer vat, Z stage, etc., as well as several alignment-assisted components, are specifically designed and machined in-house or directly purchased from McMaster-Carr. The laser source was previously purchased from Toptica, and the LED light source was purchased from Golden-Scientific, while the DMD development kit (0.95’ UV 1080 p) was previously purchased from DLi Innovation.

### 2.9 Lens Design/Mechanical Design

The lens design and optical analysis are performed with ZEMAX software. The mechanical design is performed in Autodesk Inventor.

### 2.10 System Control

Control software was developed by using LabView (National Instruments).

### 2.11 Laser Speckle Characterization

The laser speckle pattern or illumination uniformity is characterized using a beam profiler (Newport) at the plane of the polymer vat.

### 2.12 Characterization of Absorption Spectrum

Photoinitiators and photoabsorbers at 0.001% w/v were dissolved in PBS or ethanol, placed in a 4.5 mL plastic cuvette (Fisher Scientific), and then characterized using a UV–vis spectrophotometer (Thermo Fisher) to measure their absorption spectrum from 300 to 800 nm.

### 2.13 Characterization of Transparency

A UV–vis spectrophotometer (Thermal Fisher) was used to measure the transmission spectrum (400–800 nm) using 4.5 mL of 100% PEGDA polymer mixed with candidate photoabsorbers (0.01%). A DSLR camera was used to snap pictures to visualize the transparency.

## 3 Results and Discussions

### 3.1 MPS System Design

**Figure 1:**
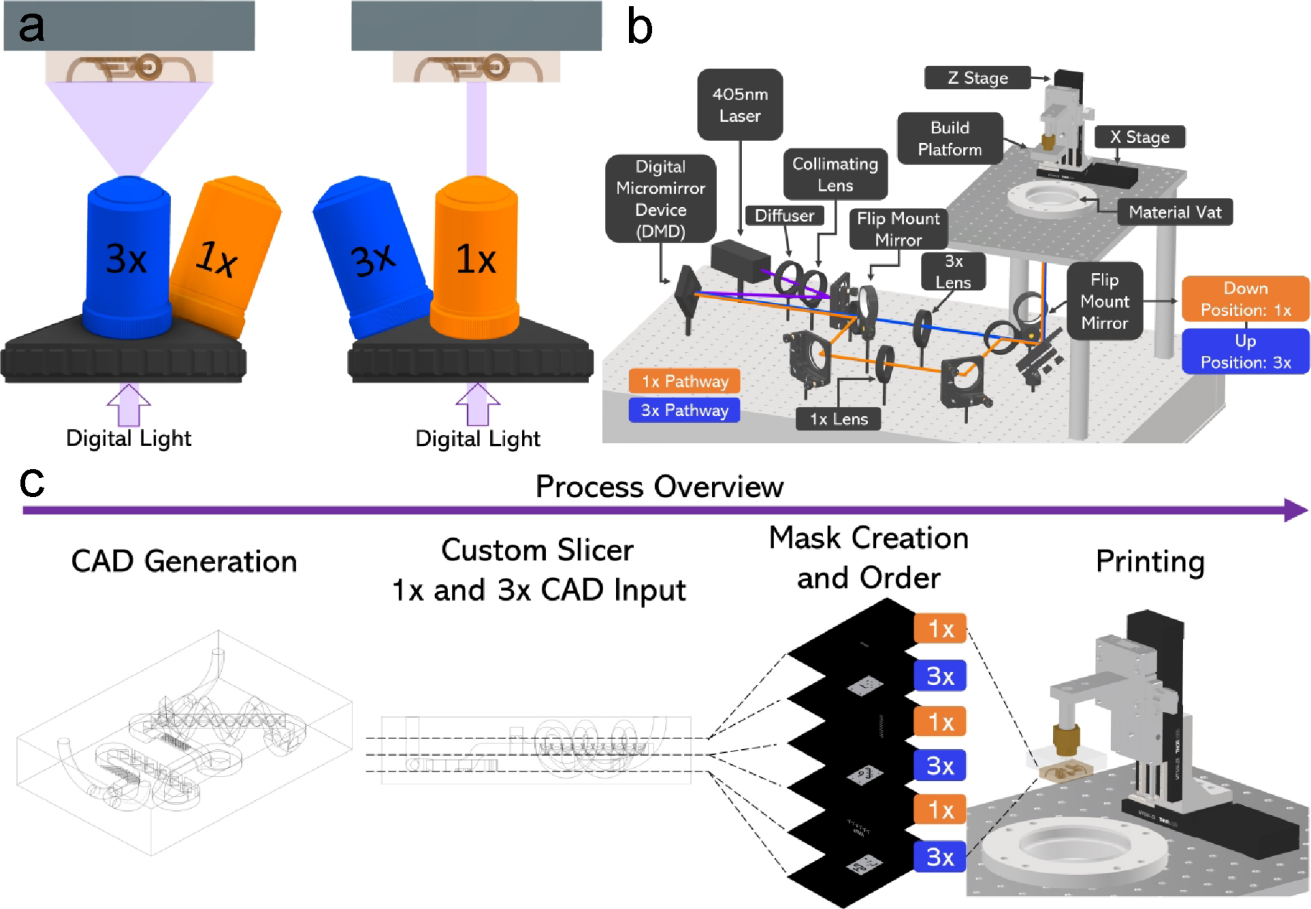
(a) Demonstration of MPS concept with two modes similar to that of multiresolution microscopy. (b) Schematic of multipath projection stereolithography (MPS) printer setup. The flip mount mirrors allow for the system to switch between 1× and 3× quickly, directing the patterned light on two different pathways: 1× (orange) and 3× (blue). When the flip mount mirrors are in the down position, constructs are printed at a 1× scale and when the flip mount mirrors are in the upward position, constructs are printed at a 3× scale. (c) MPS process overview including CAD generation, 1× and 3× CAD input to custom slicer, mask creation and order, and finally, printing.

MPS is inspired from conventional microscopy, which can switch between high- and low-resolution objectives to change the image size and feature resolution, allowing multiscale imaging. MPS utilizes two optical paths that can be switched as desired to achieve the necessary print size while maintaining high resolution (Figure 1a). Using a test case of microfluidic devices, which fit on a standard cover slide, MPS is designed with two pathways named 1× and 3× to realize a maximum print area of 30 × 60 mm while maintaining the ability to print at a resolution of 12 μm.

First, we tested two illumination systems (365 nm fiber-coupled LED, 405 nm semiconductor laser) with our multiprojection system. However, LED was not selected due to the low transmission efficiency (<50%) lens used in the setup, and associated challenges related to energy efficiency and the influence of the numerical aperture (NA) (0.5) on the projection lens. Details are given in the SI. Therefore, the 405 nm laser was selected as the light source in our setup. Briefly, the laser was collimated using a plane-convex lens with a focusing length of 150 mm (Thorlabs), and an engineered diffuser (RPC photonics Inc., USA) was used to convert the Gaussian profile of the laser beam into a top-hat profile (Figure S1). This was done to obtain uniform illumination intensity before projection onto the digital micromirror device (DMD). The lens selection was based on the divergence angle of the engineered diffuser and the illumination area of the DMD (25.4 mm). During our testing, we found that laser speckle, a common problem due to the coherence property of the laser, negatively affects the illumination uniformity. This issue was solved by designing and building a setup to rotate the diffuser and obtain illumination uniformity greater than 85%.

Here the system resolution, the smallest distance between two features, is largely designed based on the DMD micromirror size (∼10 μm, with a gap of 1 μm between mirrors). We chose a smaller numerical aperture (NA, 0.04) in both the illumination and projection setups to have sufficient depth of focus and minimize errors in opto-mechanical alignments between the Z stage and the bottom of the vat. Precision stages can resolve this issue; however, our choice of having a depth of focus over 200 μm was motivated by lowering costs and reducing the complexity. In our case, the maximum distortion across the field of view at 12 mm is less than one pixel of DMD (10.8 μm), which is less than 0.1%. For two adjacent pixels in DMD to be resolved at the image plane, the modulation transfer function or MTF@ 50lp should be more than 0.5 for 1× mode while MTF@20lp should be more than 0.5 for 3× mode. System analysis, as performed by ZEMAX, showed that both modes reached their diffraction limits. The SI shows specifics about the 2D layout of both optical systems, optimized using three main fields of view (FOV = 0, 0.707, 1). Results show that the (i) root mean square (RMS) spot radius at all FOVs is less than the airy radius (18.41 μm) showing that the system performance reached its diffraction limits, (ii) maximum distortion across field of views is less than 0.1%, and (iii) MTF at all FOVs remains under the diffraction limit; for 1× mode, MTF at full field of view remains less than 0.5. Figures S1–S3 provide additional specifics about the laser illumination system.

Thus, the final setup consists of a DMD, an engineered diffuser, a 405 nm CW laser, lenses for 1× and 3× modes, two flip mount mirrors, and X and Z stages (Figure 1b). Light irradiated from the laser was diffused, creating a uniform intensity distribution, and further collimated by illumination optics before directing it onto the DMD. The DMD used in this system consists of a 1920 × 1080 array of micromirrors with a single-pixel resolution of 10.8 μm. Following the DMD, the laser path can be directed onto two different pathways, 1× (Orange) and 3× (Blue) based on the position of two flip mount mirrors (down: 1×, up: 3×). Both pathways are directed upward by a 45° mirror toward the material vat, where material can be placed for printing. On the build platform, there is an X and Z stage to allow layer-by-layer printing in the Z and movement of 1× features in the X direction. MPS uses a simple process, beginning with a CAD generation followed by a custom MATLAB 3D slicer, which provides mask output in the correct ordering between 1× and 3× (Figure 1c).

### 3.2 Automation of MPS

To minimize alignment errors and increase repeatability, we automated both the design and printing aspects of MPS. This automation process is explained in Figure S4. Briefly, 3D CAD models, designed using Autodesk Inventor, contain macroscale and microscale features to be printed via 3× and 1× modes of MPS respectively. A custom 3D slicer developed in MATLAB was used to generate image files for both modes. The process flow starts with the user selection of CAD files to be printed with 1× and 3× modes, followed by choosing the layer heights for each mode and the layer number where the modes will be switched from 3× to 1× mode with flip mount mirror positions for each mode. Since design trade-offs make perfect alignment between modes challenging, simple image processing, such as adding an image offset, can be used to compensate for any alignment errors. MPS can also be operated in individual 3× or 1× modes. A graphical user interface (GUI) is used to monitor and control various aspects of MPS such as stage position, DMD parameters, print duration, layer heights for each mode, detachment distance, display mask images, mirror position, and other things. Before printing, the stage is lowered in a resin-filled PDMS vat to set the start position and other parameters related to laser power, image files, stage, and number of layers. A step-by-step process flow, and associated control algorithm files are provided in the SI, Section 3.

### 3.3 Characterization of MPS

**Figure 2:**
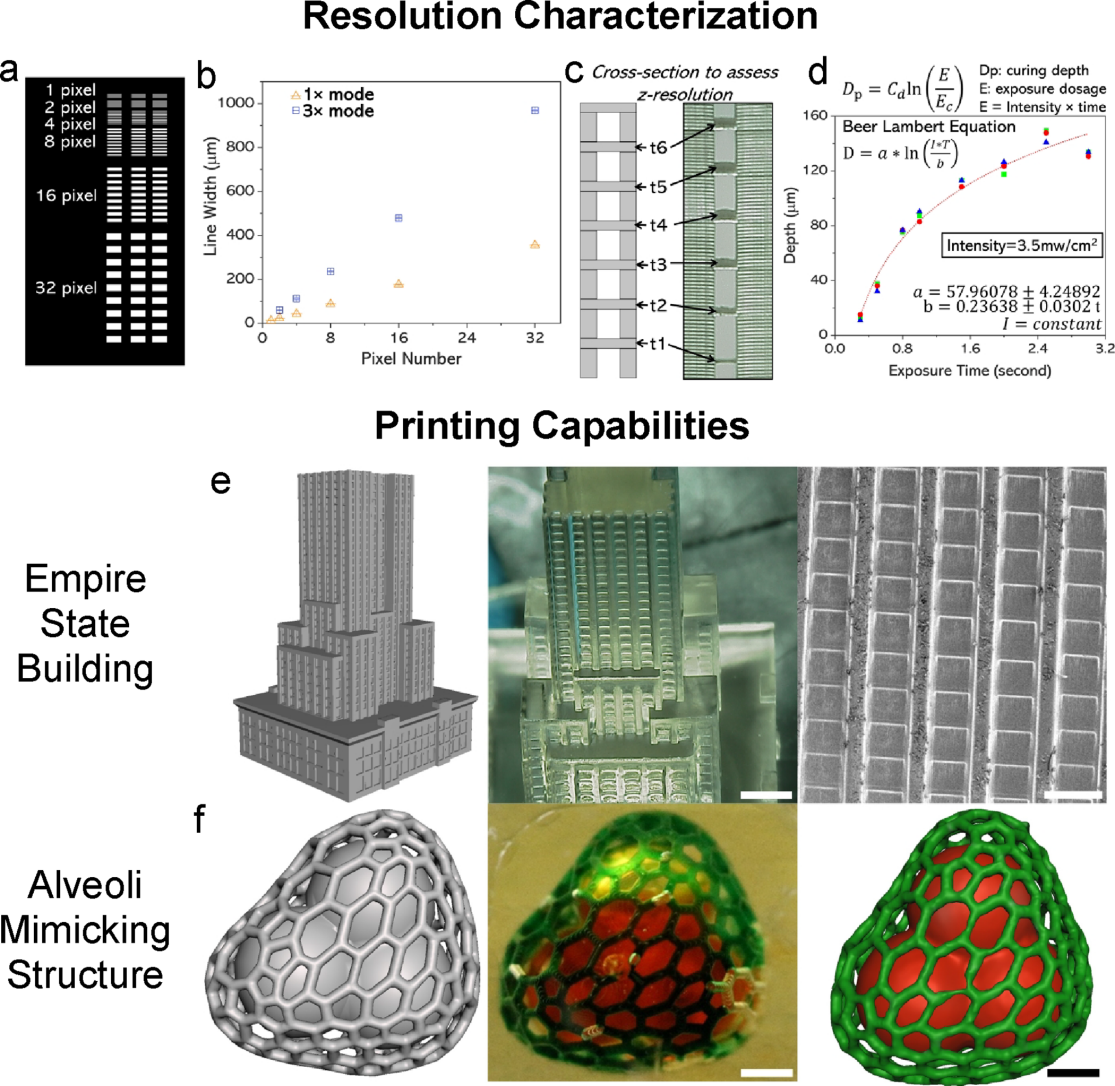
(a) Line pattern design varying from 1 to 32 pixels for XY-resolution characterization. (b) Measured line widths plotted vs pixel number show 1× resolution of 12.93 ± 1.32 μm and 3× resolution of 30.13 ± 2.09 μm. (c) Z-resolution characterization using ladder structure was completed by varying exposure time while maintaining constant light intensity. (d) Ladder results fitted using the Beer–Lambert Equation. (e) Empire State Building CAD, printed structure imaged under HIROX, and SEM image. Scale bars are 2 mm and 500 μm, respectively. (f) Alveoli model, including two individual interconnected structures CAD, printed alveoli structure imaged under HIROX, and microCT reconstruction of construct. Scale bars are 1 mm.

Resin selection remains a difficult challenge for any additive manufacturing project. In this work, we were most focused on the rapid iteration of microfluidic devices; therefore, a reliable robust material was required. Resin preparation was performed with this in mind, and the formulation was identified to include PEGDA (250 Da) as the base material, Irgacure 819 as the photoinitiator, and ITX as the photoabsorber (Figure S5). This formulation was used in the remainder of the work. To characterize the resolution of MPS, the lateral resolution was examined first. Digital masks of line patterns designed with pixel numbers varying from 1 to 32 pixels were printed using both 1× and 3× modes individually (Figure 2a) and a digital microscope (HIROX, Japan) was used to measure the line widths, showing XY-resolutions for 1× and 3× optical paths to be 12.93 ± 1.32 and 30.13 ± 2.09 μm respectively, giving an actual magnification ratio of approximately 2.8 (Figure 2b). As previously mentioned, the DMD used in this system has an array of micromirrors with a single-pixel resolution of 10.8 μm, which would represent the best theoretical lateral resolution at a 1× magnification. Analogously, at a 3× magnification, we would expect the best theoretical lateral resolution to be 32.4 μm. Z-resolution was examined next by controlling the exposure dose, a function of light intensity and time. A ladder structure was printed with varying exposure times while maintaining a constant exposure intensity of 3.5 mW/cm^2^ (Figure 2c). Results were fitted using the Beer–Lambert Equation, showing Z-resolution variation between 12.68 and 132.75 μm for an exposure time range of 0.3 to 3 s (Figure 2d). Based on these results, we choose a layer height of 50 μm using an exposure time of 0.8 s per layer for 1×. In order to maintain the same layer height, this experiment was repeated for 3× mode, where the laser intensity remained the same, but the tested exposure time range increased (larger area, lower exposure intensity, longer exposure required). As expected, a longer exposure time of 3 s was chosen based on the graphed curve. Layer height can be varied by modifying the exposure time in the GUI described earlier while maintaining a constant light intensity. For this material formulation, we found no delaminations at a layer height of 50 μm using both modes. Further material optimization could be performed to decrease the layer height more, but for this work, a constant layer height of 50 μm was maintained.

The capability of printing complex designs was tested using the 3× mode of MPS. First, the Empire State Building was modeled (Figure 2e) and printed. Images taken using HIROX distinguish microscale windows of building and scanning electron microscopy (SEM, JEOL5600, Japan) images distinguish features printed using single-pixel light exposure. With an approximate volume of 1.4 cm^3^, this structure was printed in less than 5 min. Second, an alveoli-mimicking structure with complex hollow topologies was tested. A representative alveoli found in the human lungs was designed (Figure 2f) and printed. It consisted of two independent hollow features, including an interconnected air sac (red food dye) and a network of microchannels surrounding the air sac structure, representing blood capillaries (green food dye). To accurately characterize the printed construct, micro computed tomography (micro-CT 40, Scanco Medical AG, Brüttisellen, Switzerland) was performed. Results show a high printing accuracy.

### 3.4 Rapid Prototyping of Microfluidic Mixers

**Figure 3:**
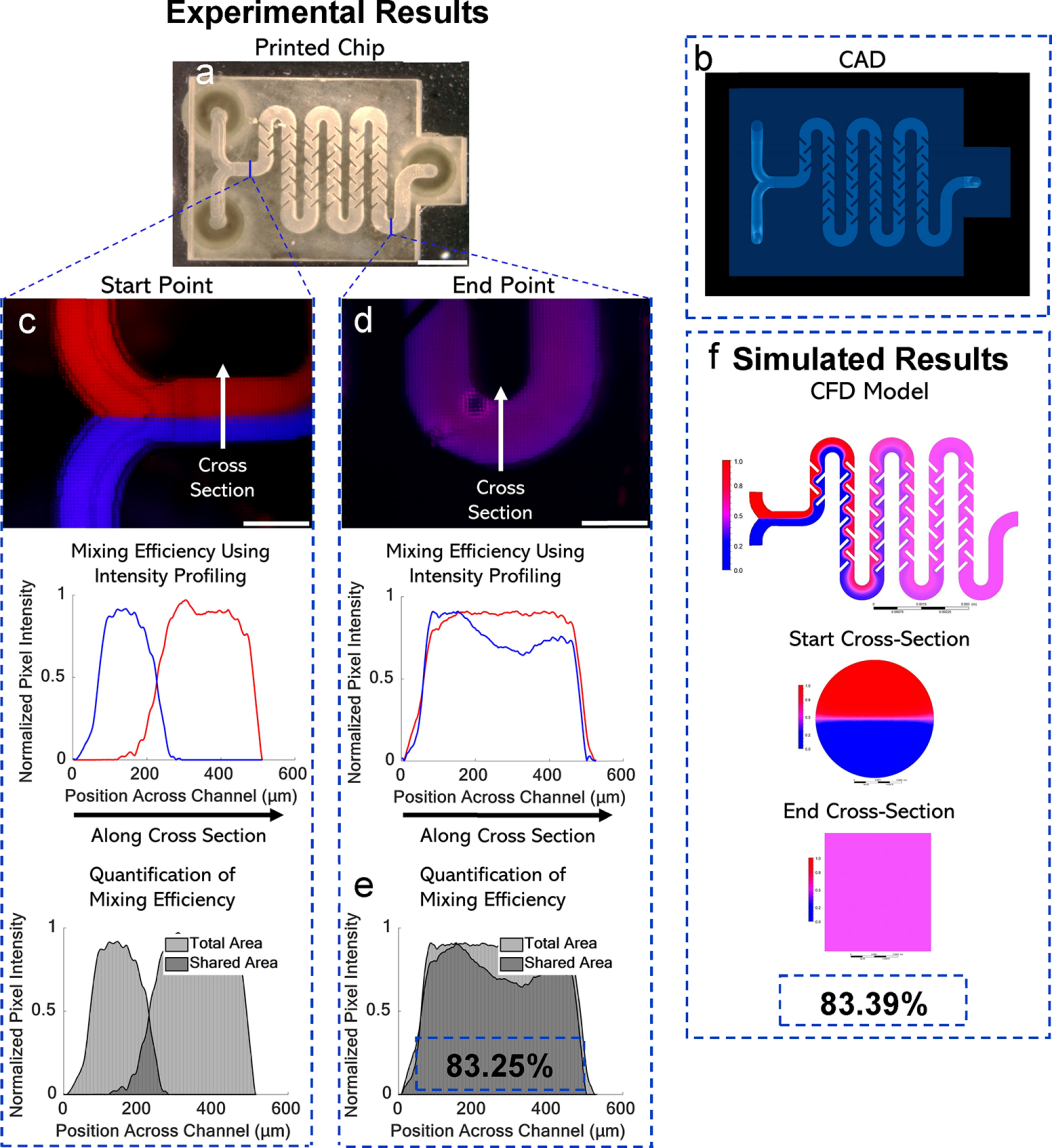
Microfluidic mixers as an example of the rapid iteration of printable microfluidic devices. (a) Fixed solid wall printed result, top view. Scale bar is 2.5 mm. (b) Top view of a fixed solid wall CAD model. (c) Fluorescence image of the start position and mixing efficiency graphs using intensity profiling. Scale bar is 500 μm. (d) Fluorescence image of the end position with mixing efficiency graphs. Scale bar is 500 μm. (e) Quantification of mixing efficiency using normalized pixel intensity across the channel position. (f) CFD results include top view and start/end cross section views.

Microfluidic mixers were chosen as a test case to demonstrate MPS’s capability of printing microscale features in any defined location within a macroscale device. With insight from the literature, three microfluidic mixer devices were designed and printed using MPS. In the field, the three most common mixers utilized included 3D spiral fins forcing fluid horizontally and vertically, fixed solid wall fins where fluid is forced through a pathway, and a herringbone pattern where fluid flows over the top.^35−38^ Additionally, most mixers followed a serpentine pattern to maximize channel length and mixing efficiency within fixed chip size. With these specifics in mind, the first mixer was designed with a 500 μm wide serpentine channel within a 12 mm × 8 mm microfluidic chip. This included two inlets and one outlet. The channels were 400 μm in height, and this design was drawn with 100 μm fixed solid fin walls at 45°. This allowed for a 300 μm opening for fluid flow between the fins. The overall chip and serpentine were printed in under 10 min using 3× mode and the fixed solid fin walls were printed using 1× mode of MPS (Figure 3a). To maintain consistency, features that were 100 μm or less were printed with 1× mode, and anything larger was done with 3× mode. Feature size discretion was addressed by the automated 3D slicer, and respective masks were formed as a result. The top view of the CAD model is shown (Figure 3b). We included computational modeling and experimental results to have a complete approach that is tunable for different applications. For this first mixer, experimental analysis was performed first to validate custom computational fluid dynamics (CFD) algorithm data to develop a predictable model. CFD analysis was performed using ANSYS. To certify the laminar flow conditions, we conducted a flow simulation (without diffusion) for each case to calculate the maximum velocity and the corresponding Reynolds number in the channel (SI Section 5). To assess mixing efficiency experimentally, fluorescent dyes were chosen.^38^ 150 kDa FITC-dextran and 70 kDa Rhodamine-dextran were flowed in each inlet at 5 μL/min, controlled by a syringe pump. Fluorescence images were acquired throughout the chip; the inlet was chosen as the baseline for mixing efficiency, (Figure 3c). The mixing ratio was calculated in MATLAB by computing the percent overlap between the normalized fluorescence intensity profiles at the start and end of the flow channel.^38^ The image of the endpoint is shown with mixing ratio graphs (Figure 3d). The final mixing ratio of this mixer was determined to be 83.25% (Figure 3e). CFD results of the top view of the channel and cross sections of the start and end sections of the channel are shown (Figure 3f). Mixing efficiency was determined to be 83.39% using methods described in Figures S6–S9. Additional images including an isometric CAD view, no roof internal view of fin design, fluorescent image from the middle section, and SEM characterization can be seen in Figure S16a. Experimental results align well with the CFD mixing efficiency results, further emphasizing the print quality and success of the MPS system for this application.

**Figure 4:**
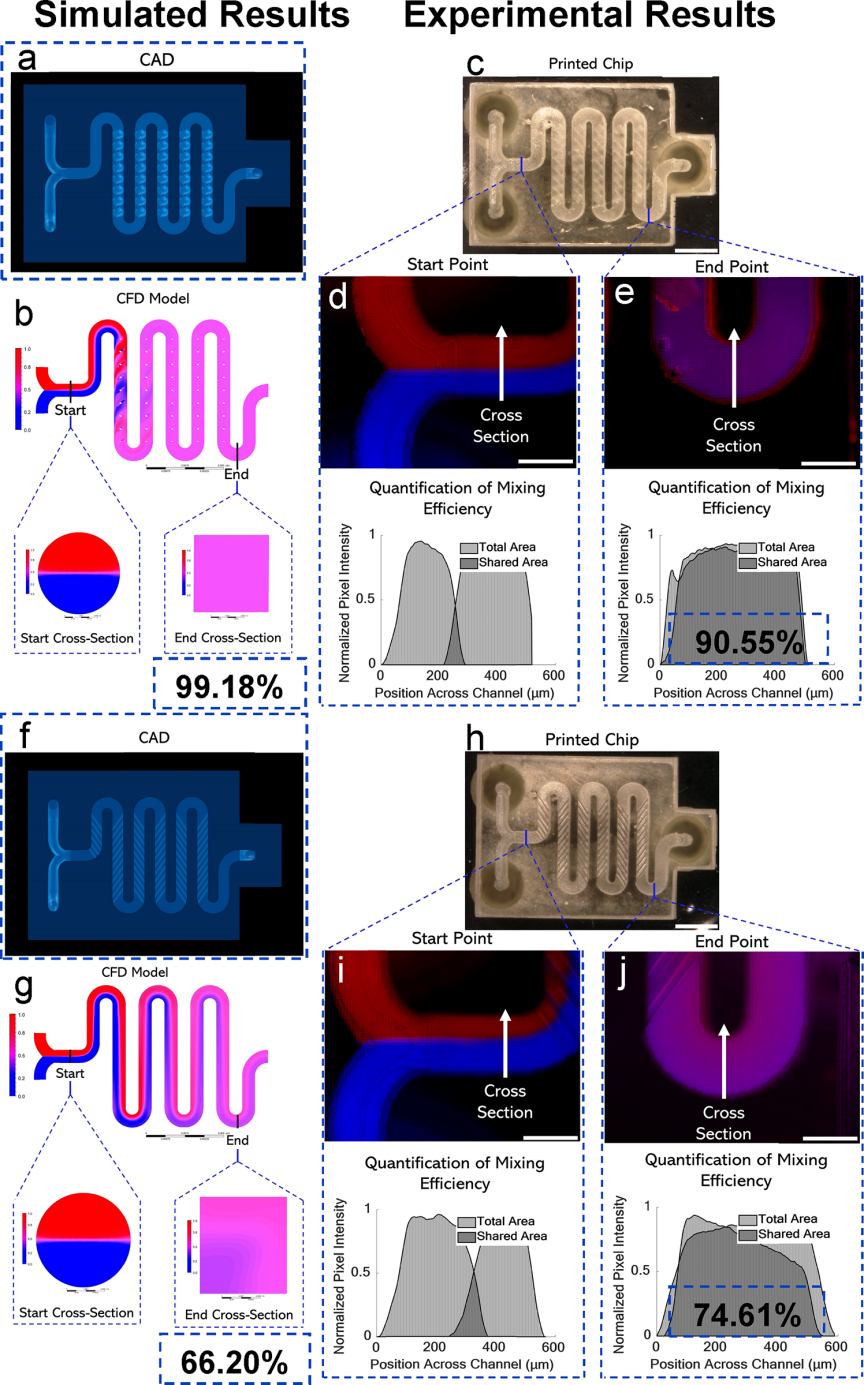
(a) Top view of the 3D spiral CAD model. (b) CFD results include top view and start/end cross section views. (c) 3D spiral printed result, top view. Scale bar is 2.5 mm. (d) Fluorescence image of start position and mixing efficiency graph using intensity profiling. Scale bar is 500 μm. (e) Fluorescence image of end position with mixing efficiency graph illustrating quantification of mixing efficiency using normalized pixel intensity across the channel position. Scale bar is 500 μm. (f) Top view of the herringbone CAD model. (g) CFD results including top view and start/end cross section views. (h) Herringbone printed result, top view. Scale bar is 2.5 mm. (i) Fluorescence image of start position and mixing efficiency graph using intensity profiling. Scale bar is 500 μm. (j) Fluorescence image of end position with mixing efficiency graph. Scale bar is 500 μm.

For validation of our rapidly iterative printing approach, two additional microfluidic mixers were designed with the same overall chip size, serpentine channel width, and height, but the mixing feature to be printed by 1× was changed. CAD design to the printed structure can be completed in under 2 h. The second mixer was designed with 100 μm wide 3D spiral fins, using a 1440° twist, creating 8 rotations per straight section of the serpentine (Figure 4a). Here, the CFD model, validated using the fixed solid design, was used to calculate mixing efficiency before printing the device with MPS (Figure 4b). Mixing efficiency was determined to be 99.18% from CFD analysis using the methods illustrated in Figures S10–S12. Again, the chip was printed with 3× and the 3D spiral fins with 1× (Figure 4c). Fluorescence images and mixing efficiency are illustrated in (Figure 4d,e). The final mixing ratio of the second mixing design was determined to be 90.55%. Additional images including an isometric CAD view, no roof internal view of the spiral section, fluorescent image from the middle section, and SEM characterization can be seen in Figure S16b. Experimental and computational results of mixing efficiency match well.

The third mixer was also designed with the same chip size and serpentine characteristics and included a herringbone pattern on the bottom of the channels. The serpentine pattern was printed with a 100 μm height, 100 μm gap between each fin, and an experimentally determined 35.6° angle (Figure 4f).^35^ CFD analysis determined mixing efficiency to be 66.20% (Figure 4g) using the methods illustrated in Figures S13–15. The printed chip result is shown in the top view (Figure 4h). Top-view fluorescence images from the start and end points are shown along with mixing efficiency graphs (Figure 4i). For the herringbone fin design, the mixing efficiency was shown experimentally to be 74.61%. Similar images including an isometric CAD view, no roof internal view of herringbone pattern lining the bottom of channels, fluorescence top view image, and SEM characterization can be seen in Figure S16c. Overall, experimental results from all three fin designs showed consistency with the CFD mixing efficiency results.

### 3.5 Complex 3D Microfluidic Mixers

**Figure 5:**
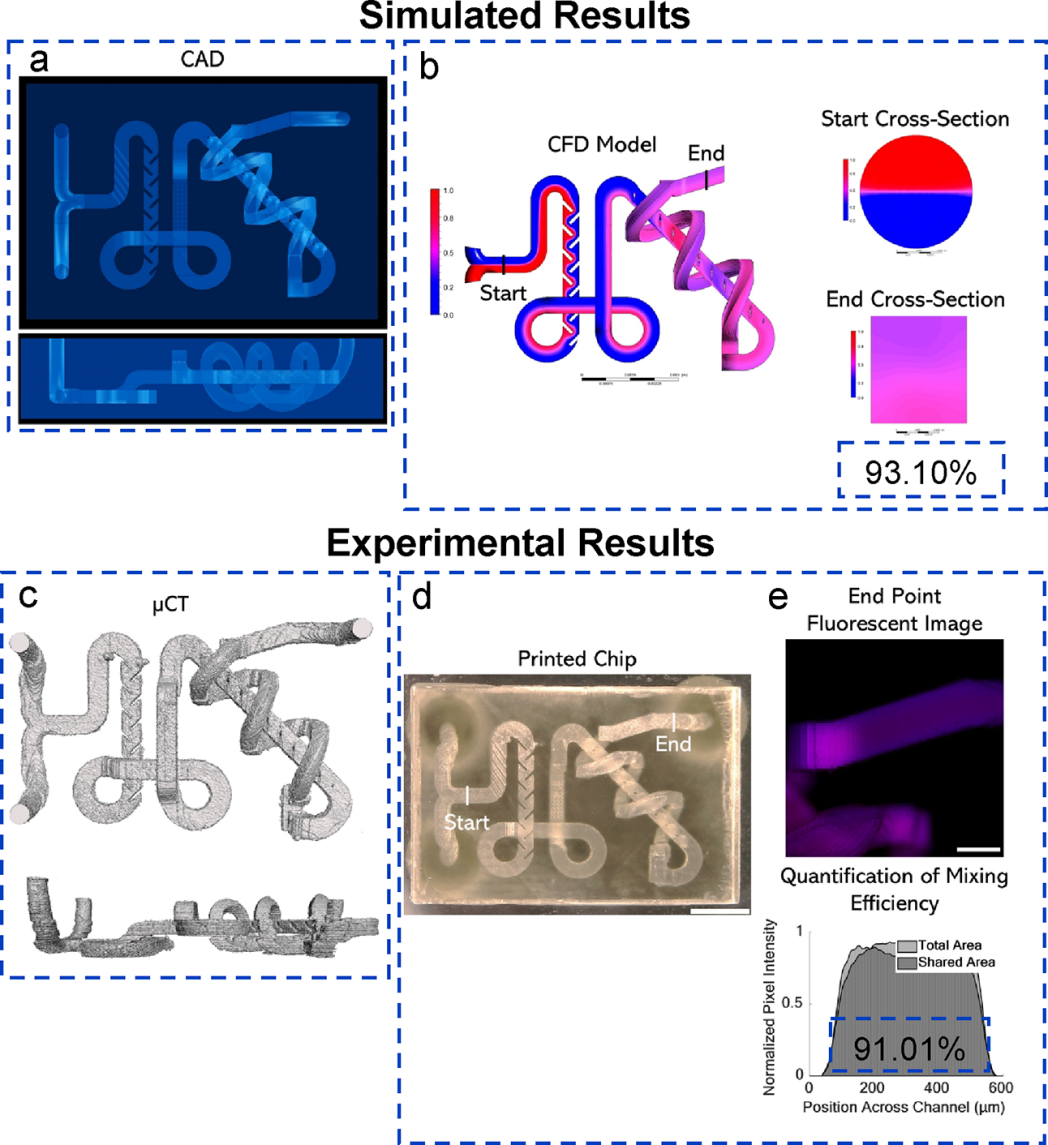
(a) CAD of a complex 3D microfluidic mixer including top and side view. Features on three planes include herringbone, fixed solid fin wall, micro dots, and 3D spiral. (b) CFD results include the top view and start/end cross section views. (c) microCT reconstruction of a microfluidic mixer with the same model views. (d) Printed result, top view. Scale bar is 2.5 mm. (e) Fluorescence image of end position with the mixing efficiency graph. Scale bar is 500 μm.

Using inspiration from the features designed in the previous mixers, two complex 3D mixers were designed to further highlight the unique capabilities of MPS, particularly its ability to print complex structures in multiple locations in 3D. In the first CAD, channels were designed to flow and overlap on three planes. On each plane, a different mixing feature design was incorporated. The first utilizes a herringbone design and a fixed solid fin wall structure. The second and third planes feature a 3D spiral design inside the channel and an array of microdots, respectively. Again, the top and side views of the 3D CAD are shown (Figure 5a). The channels overlap each other in multiple planes, including a spiral around a section of the channel. CFD highlighted the efficiency of the design at 93.10% (Figure 5b). Furthermore, microCT was performed for printing validation, and the results are shown in (Figure 5c). Finally, the printed result is shown from a top view with a final mixing efficiency of 91.01%, (Figure 5d).

In the second complex CAD design, channels were designed to flow and overlap on two planes. On the bottom of channels throughout the chip, the same herringbone pattern was used as the mixing design. The top and side view of the 3D CAD is shown (Figure S17a). CFD analysis was performed preprinting to allow for further optimization of the design. The end cross-section result is shown, highlighting the high efficiency of the design at 98.31% (Figure S17b). To accurately characterize the printed mixers, we used microCT. Results shown in the same views exhibit excellent mimicry of the original CAD design (Figure S17c). The printed result is shown from a top view and fluorescent mixing efficiency is highlighted at 90.18% (Figure S17d). A summary of all chip mixing efficiencies is shown in Figure S18.

### 3.6 Other Microfluidic Devices Using MPS

**Figure 6:**
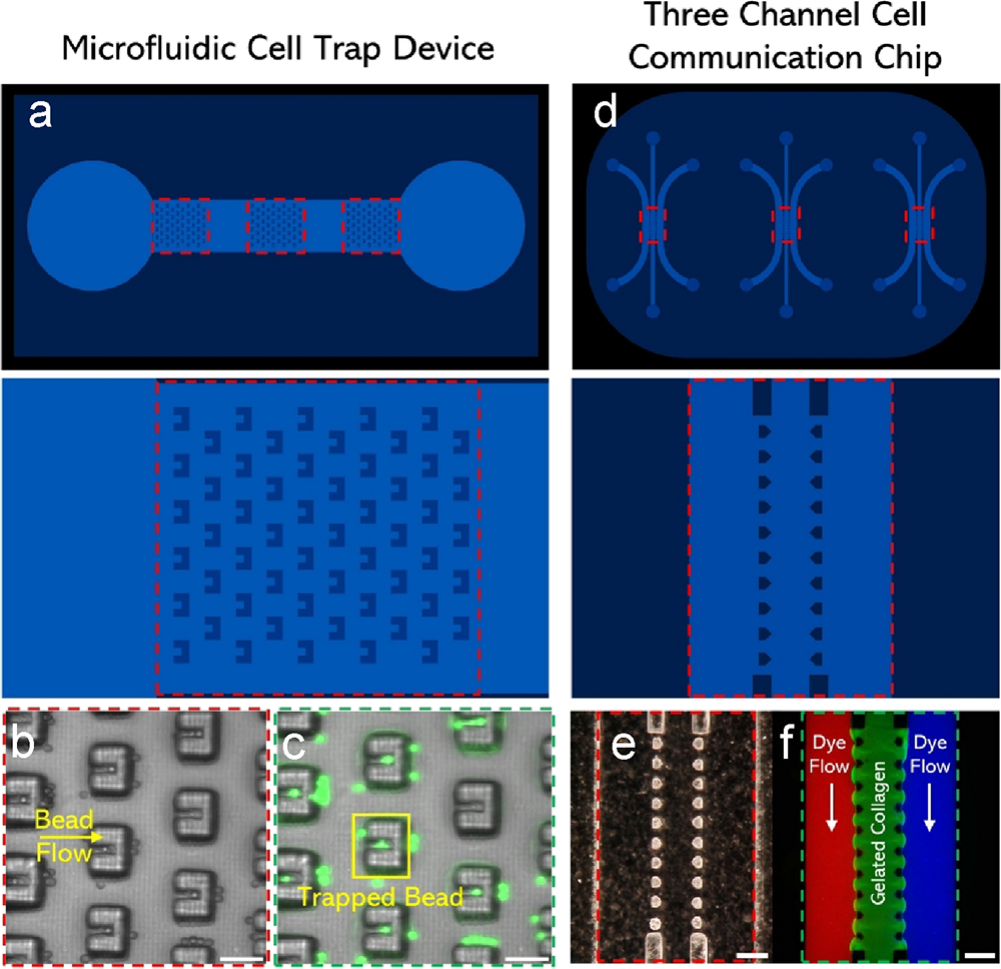
(a) CAD of a large-scale microfluidic cell trap device. Three sets of cell trap arrays are printed by 1× on top of a 3× printed base. (b) Brightfield image of microparticle solution flowing through chip; microparticles are being stopped by traps. Scale bar is 100 μm. (c) Fluorescence image of microparticles seen in traps. Scale bar is 100 μm. (d) Three-channel cell communication microfluidic chip in triplicate (high-throughput). Scale bar: 2 mm. (e) HIROX image of microposts created by 1× in between the three channels. Scale bar is 300 μm. (f) Central channel filled with fluorescent collagen and outside channels filled with microparticle solution. Scale bar is 300 μm.

To demonstrate the feasibility of using MPS to print large-scale devices with high resolution, we printed a simple cell-trapping microfluidic device using both 1× and 3× modes. The microfluidic chip base, 40 × 20 mm, was printed using 3× mode with three microtrap arrays embedded within the device printed using 1× mode (Figure 6a). The height of the channel was 500 μm, while the height of the microtraps was 100 μm. Postprinting, a fluorescent microparticle solution (diameter = 18.67 μm, 10% v/v) was perfused with the central channel, and a fluorescence microscope (Nikon, Japan) was used to capture images (Figure 6b,c). Printed microtraps, with a width of 60 μm and a trapping opening of ∼20–22 μm were able to trap single microparticles. Some of the traps were also able to trap more than one microparticle. These results demonstrate the potential of such a multiscale printer to rapidly print microfluidic devices for a range of applications.

A second microfluidic device, a three-channel chip commonly used in 3D cell culture and organ-on-chip applications, was designed and printed using MPS. Three sets of the channel designs were printed within a single large-scale chip (Figure S19) using the 3× mode, (Figure 6d), while micropost arrays were printed using the 1× mode of MPS (Figure 6e). In a typical application, an extracellular matrix or hydrogel solution perfused within the central chamber does not leak into the side channels. To demonstrate this, a 2% gelatin and 5% 2000 kDa FITC-dextran solution was flowed into the central channel and allowed to thermally cross-link and solidify, before perfusing another fluorescent solution (150 kDa FITC-dextran) into the side channels (Figure 6f). Fluorescence microscopy images demonstrate no fluidic leakage between the three channels. These results highlight the unique ability of MPS to create high-resolution microstructures in any location within a macroscale printed construct.

## 4 Conclusions

This study reported an alternative approach to fabricating multiscale microfluidic devices by combining a high-resolution and low-resolution mode into a single printing system. It overcomes certain trade-offs found in the field between printing resolution and printing area. Conventional 3D printing methods, FDM for instance, have the ability to create large-scale devices; however, lateral resolution is limited to ∼100 μm, which is not sufficient for high-quality microfluidic devices. Researchers have turned to VPP as a promising alternative, specifically DLP-VPP where higher resolution (<50 μm) has been extensively reported. The major limitation of DLP-VPP is that its projection (build) area is inversely proportional to its feature resolution, which limits the creation of larger-scale devices with high feature resolution. MPS utilizes DLP-VPP with inspiration from microscopy with multiple quick-change magnifications to overcome the aforementioned limitations. MPS does not come without its own limitations, but future work gives a promising path to address them. MPS is a powerful technology that can be applied to scales even larger or more importantly, smaller. With its concept demonstrated with 1× and 3×, there is an extendable capability for the system to be built with additional pathways including a 0.1× for even higher resolution features and/or 6× for larger-scale devices. Though the existing MPS system utilized a single optimized material for microfluidic devices, as a DLP platform, it is inherently compatible with a diverse range of photo-cross-linkable materials, which further extend its breadth of potential applications. One of the most challenging aspects of MPS is the physical alignment of the multiple pathways. It is difficult to attain alignment of any optical system, so an imaging processing algorithm was added within the slicer to mitigate misalignment. Utilization of different motorized optical components will be done in the future to minimize the need for such corrections. Finally, the slicer software that was developed relies on some user input to specify which features within the CAD model are to be printed with 1× and 3 ×. The existing algorithms can be augmented with more complex and intelligent detection abilities in the future. Such improvements to the print process will allow for our MPS platform to fabricate even more advanced structures for a wider variety of biomedical applications.

## Supporting Information Available

Additional details on optical setup and design, material screening process, and computational fluid dynamic experimental methods, including mixing design characteristics (PDF)
