# Supplementary material for: Multipath Projection Stereolithography for Three-Dimensional Printing Microfluidic Devices
Source: ACS Appl Mater Interfaces. 2024 Dec 3;16(50):69807–17. doi: 10.1021/acsami.4c10547 (PMC11660027; doi:10.1021/acsami.4c10547)
Supplement: Supplementary file 1 — am4c10547_si_001.pdf [file am4c10547_si_001.pdf]

Supporting Information

Multipath projection stereolithography (MPS) for 3D printing microfluidic devices

Zachary J. Geffert[1§], Zheng Xiong[1,3§], Jenna Grutzmacher[1], Maximilian Wilderman[1], Ali Mohammadi[2], Alex Filip[1,3], Zhen Li[2], Pranav Soman[1,3*]

[1]*Department of Biomedical and Chemical Engineering, Syracuse University, 900 S Crouse Avenue, Syracuse, NY 13244, United States,*

[2]*Department of Mechanical Engineering, Clemson University, 105 Sikes Hall, Clemson, SC 29634, United States,*

[3]*3D Microfluidics LLC, 5900 Strawmount Trail, Chittenango, NY 13037, United States*

[*]Email: psoman@syr.edu

[§]Z.J.G. and Z.X. contributed equally to this work

1. Laser illumination system.

A semiconductor continuous-wave laser (405 nm with 8 nm bandwidth, TOPTICA, Germany) is collimated using a plane-convex lens with focusing length 150 mm (Thorlabs). An engineered diffuser (RPC photonics Inc., USA) is used to convert the Gaussian profile of the laser beam into Top-hat profile (**Fig. S1**). This is important to obtain uniform illumination intensity before projecting onto the DMD. The lens selection was based on the divergence angle of the engineered diffuser and the illumination area of DMD (25.4 mm). The focusing length of collimation lens is given by

$$\tan \phi = R/f$$

where ϕ is the divergence angle of the engineered diffuser (which is 5°), R is 0.5D the radius of lens aperture *(which should be larger than 12 mm or ½ the size of DMD)*, and f is the focusing length of the collimation lens.

Problems encountered. Laser speckle, a common problem due to coherence property of the laser, negatively affects the illumination uniformity. This issue was solved by designing and building a setup to rotate the diffuser and obtain an illumination uniformity greater than 85%. To achieve high energy efficiency, the NA of projection system should be equal to or greater than the NA of the illumination system. However, higher NA will induce small depth of focus, which contributes to greater opto-mechanical misalignment. To solve this challenge, we choose laser illumination system with a smaller NA and thus decrease the strict requirement of NA for the projection lenses used in the system.

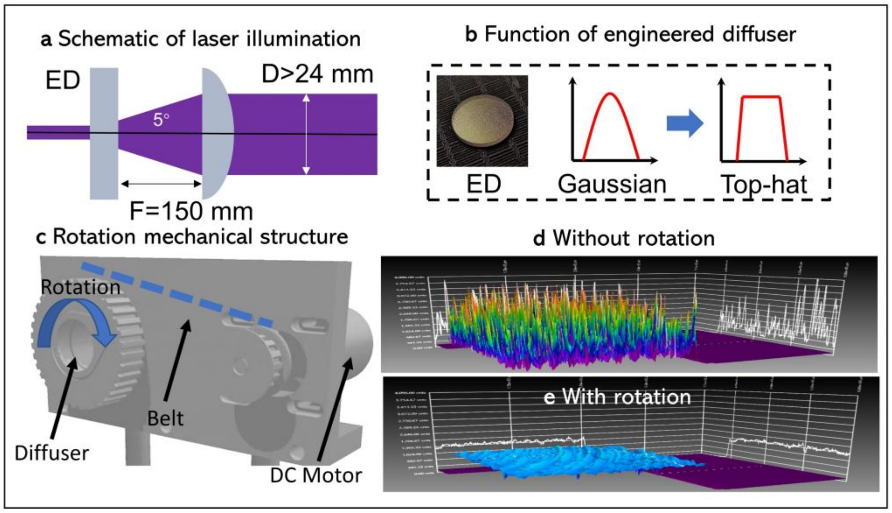

Figure S1. *Laser illumination system.* ***a*** *Schematic of optical setup.* ***b*** *Function of engineered diffuser converting Gaussian profile of laser into Top-hat profile.* ***c*** *Rotating mechanical structure to generate uniform laser speckle.* ***d*** *Laser speckle profile with and without rotation.*

2. Multi-path projection systems (1× and 3×) used in MPS

We have designed a projection system with two optical paths, 1× mode and 3× mode. 1× mode has a print area of 10 mm x 20 mm with a print resolution of **~12 µm** while 3× mode has a print area of 30 mm x 60 mm with a print resolution of **~32 µm**. Zemax was used to design the projection lenses based on several specifications, as discussed below.

Numerical aperture. NA of the projection system determines the resolution and the depth of the focus based on the following equations:

System resolution, R=kλ/**NA**

Where R is the system resolution, k is the lithography parameter, here, we defined as 0.5, NA is numerical aperture.

Depth of Focus, DOF~=kλ/**NA**^{2}

where NA=nsin ϕ, DOF is the depth of focus, λ is the wavelength of the light source, NA is the numerical aperture of the system, ϕ is the divergence angle of the engineered diffuser.

(**Figure S2a**) System Resolution. Digital Micromirror Device (DMD) used in the setup consists of 1920 × 1080 micromirror array; size of each micromirror is 10 µm with inter-mirror gap of 1 µm. The resolution of the projection system should be low enough to not resolve the inter-mirror gap but high enough to resolve single micromirror (pixels), i.e, 10 µm>R>1 µm.

(**Figure S2b**) Depth of focus (DOF). DOF is the axial depth of the space on both sides of the image plane within which the image appears acceptably sharp. The stage, required to print large sizes, was found to be difficult to align with bottom surface of the vat; even microscale tilts of the stage resulted in opto-mechanical misalignment of ~100 µm **(Fig. S2)**. Precision stages and components, needed to address this issue, were found to be prohibitively expensive. As a result, a DOF of 200 µm was finalized for this design. Based on this, NA will be 0.04. According to our system design, NA=nsinϕ=0.04 corresponds to divergence angle ϕ=2.3°. This divergence angle requires a high

collimation; this is another reason why we choose the laser source in the illumination system as compared to LED.

Distortion describes the magnification in the image plane changes across the whole field of view. Since the maximum distortion at full field of view@12 mm should be less than one pixel of DMD (10.8 µm), the distortion at full-field of view should be less than 0.1%.

Distortion, Dy = (yp - y0)/y0 %

where Dy is the distortion, yp is the actual image height (in our case, it is ~12 mm ± 10.8 µm, y0 is the ideal image height *(in our case, it is 12 mm)*.

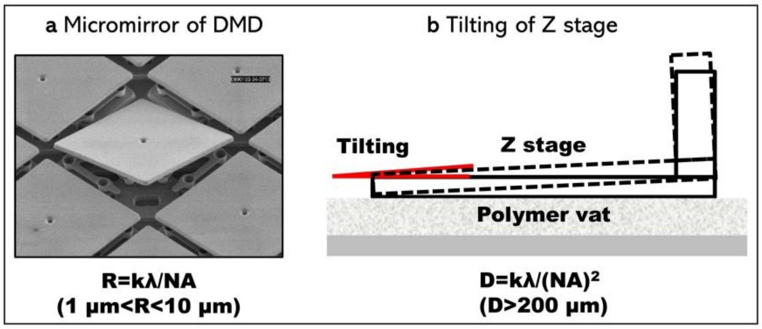

Figure S2. *Schematic showing alignment challenges. NA is based on resolution and depth of focus.* ***a*** *Image of DMD micromirrors highlighting resolution and NA equation.* ***b*** *Image depicting tilting of the Z stage emphasizing DOF and NA.*

The modulation transfer function (MTF) determines how much contrast in the original object (DMD) is maintained at the image plane. To resolve two adjacent pixels in DMD at the image plane. Therefore, MTF @ 50l p should be more than 0.5 for 1× optical path, and MTF @20l p should be more than 0.5 for 3× optical path.

1/(2d) >0.5

where d is the pixel size at the image plane. For 1×, d = 10.8 µm. For 3×, d = 32.4 µm.

System design and analysis using Zemax. With these system specifications, we set the merit function in Zemax with optimization of primary aberration and the control of the magnification ratio. **Figure S3a** demonstrates the 2D layout of the optical systems. Optimization was carried out with three main field of view (FOV=0, 0.707, 1). So, the three color (Red, Green, Blue) represents the light beam from these FOVs. Zeemax was used to run system analysis, and results show that the performance for both 3× and 1× modes reach close to their diffraction limitation. *(close to ideal results)*

For 3× mode, we choose a plane-convex lens with *F=300 mm* and a tube lens with *F=200 mm*. For 1× mode, we choose a achromatic doublet with *F=200 mm* and a tube lens with *F=200 mm*. We simulated several off-shelf lens choices and found that tube lens has the better imaging performance across all the field of view due to its overall aberretion correction than standard achromats.

Spot diagram. Spot diagram presents the imaging performance of single dot at different field of views (FOVs). **Figure S3b** presents the spot diagram for the 3 FOVs. The three colors in this diagram presents 3 main wavelengths (0.405 µm, 0.407 µm, 0.409 µm). The RMS spot radius

listed at the 3 FOVs are less than airy radius **(18.41 µm)**; *airy radius represents an ideal system under diffraction limitation*. The airy radius is the black circle in **Fig. S3c**. This demonstrates that the system performance have reached its diffraction limitation.

Distortion diagram. The X axis of the distortion diagram is the percent distortion. The Y axis of the diagram is the FOV. Results in **Fig. S3d** show that maximum distortion is at the full field of view, which is still less than 0.1%; this meets our target specifications.

MTF diagram. MTF is an important index to characterize system performance. Regularly, we quantify the performance using line patterns with different spatial frequency and characterize its image contrast at the final image plane. In **Fig. S3d**, the X axis of the MTF diagram represents spatial frequency per cycle of line pattern. The Y axis of the diagram represents the modulus of the optical transfer function, which is image contrast. The three colors in this diagram represents 3 FOVs. We can see that the MTF at all FOVs is close to the ideal performance under diffraction limitation which is the dashed black lines in the figures. Even though MTF for 1× system at full field of view is still a bit less than 0.5, the system printing performance comes close to the ideal which is promising.

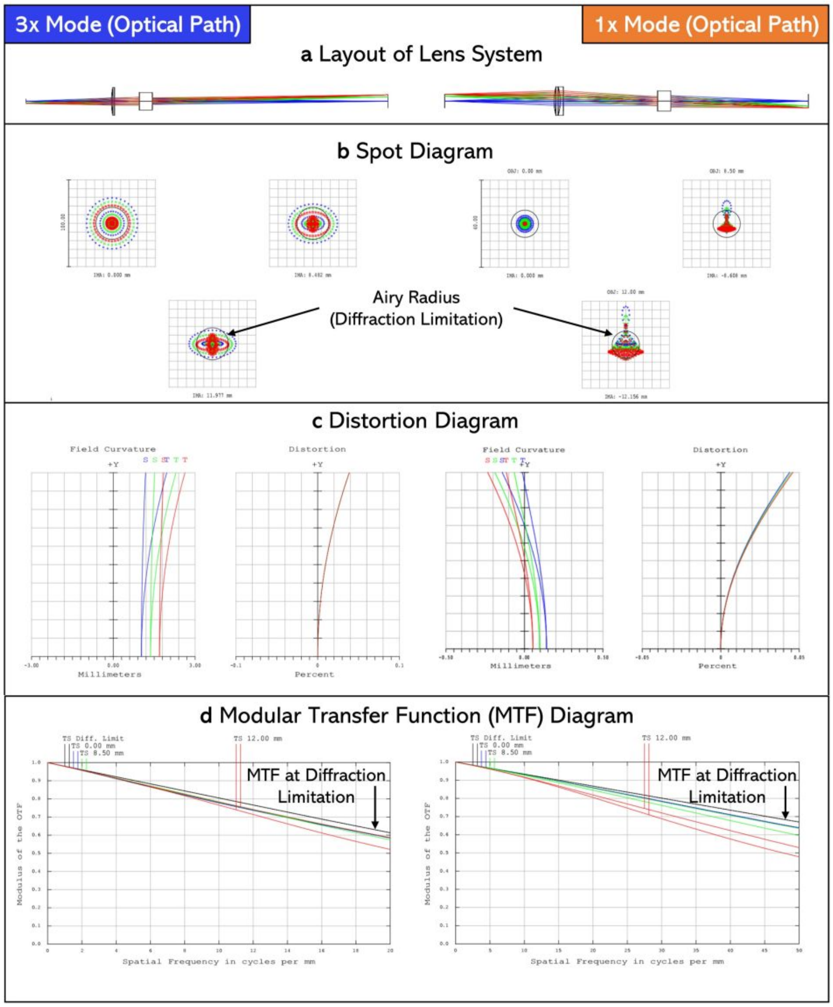

Figure S3. *Projection lenses for both 3× and 1× modes of MPS optimized using Zemax simulations.* ***a*** *Lens layout of the system.* ***b*** *Spot diagram.* ***c*** *Field-curvature and distortion diagram.* ***d*** *MTF diagram.*

3. MPS Printing process

Custom slicer. MATLAB was used to develop a custom 3D slicer to slice CAD files layer by layer into respective individual image files. The slicer was designed as a function to be run in one easy step. Once run, the user is directed through a series of prompts. This slicer can be used for all 3 modes of our printer, 1× individually, 3× individually, and 1× and 3× combined. The first prompt asks the user if they want to print with the combination mode. If yes, it then prompts the user to select each CAD file (.stl format) for 1× and 3× mode. The user is prompted after this step to select the final output folder for the images. The next prompt asks the user for the slice height for each mode in millimeters. The final step asks the user which layer of the 3× CAD the 1× features begin on. This will allow the slicer to order the images correctly in the output folder. Generally speaking, a print will have large features printed by 3× and higher resolution internal features done by 1×. Once the slicer has all the information, the slicer will individually slice each CAD file by the desired slice heights and order the images correctly based on the user input. The slicer also outputs a text file containing the flip mount mirror positions for each layer, to enable automation of the entire printing process. If the user selected no at the first step, they would be directed to use the single mode capabilities of the printer. Both 1× and 3× follow the same first 3 prompts where the user will select the desired CAD file, select the output folder for the images, and input the desired slice height. For 3× mode, the code will be complete, and the output images will be displayed for the user. At this point, the multimode and 1× individual paths have another prompt which allows for an image offset for 1×. If no offset is required, this is the final step, and the output images will be displayed for the user. The addition of a software offset to the slicer stems from perfect physical optical alignment of 1× and 3× beam paths being near impossible. Once the physical alignment is adequate, simple image processing can be used to get the alignment perfect. This process is highlighted in supplemental figures. If an image offset is needed, the user will be prompted to input an X and Y offset value in millimeters, then the output images will be displayed for the user. This is the end of the code. The current version of this slicer is version 7 and has undergone many iterations to increase the versatility of the slicer as well as meet the complex needs of the MPS printer.

Printer workflow. The MPS printer workflow is illustrated in **Fig. S4**. A few prepatory steps are performed before printing occurs. This includes loading the desired photoreactive material into the PDMS dish, lowering the stage to create an oxygen permeable 'dead-zone,' setting the mirror mount to a starting position, and inputting various printing parameters. The required parameters include laser power, the image file folder location, first layer height, first layer show time, remaining layer heights, remaining layer show times, stage velocity and acceleration, detaching distance, number of layers, and the 1× and 3× text file folder location. Now, printing is ready to begin. The DMD will be enabled and expose a UV image for the set base layer show time. Following exposure, the code will check the next layer. If the next layer is 3×, the stage will move the desired detatchment distance to allow for new material flow, then move back down the next Z layer height and expose the next layer. If the next layer is 1×, the mirrors will flip and print the 1×

layer at the same Z height. A 1 s pause is included after mirror flipping to allow for any vibrations in the mirror from the flipping motion to be become negligible. We do observe some changes in the focus over time, but the flip mounts are just one source. Following this for either, 1× or 3×, the code will look for the next image, if there is one, the checking of the next layer process will repeat. If there are no more images, then the print will be complete.

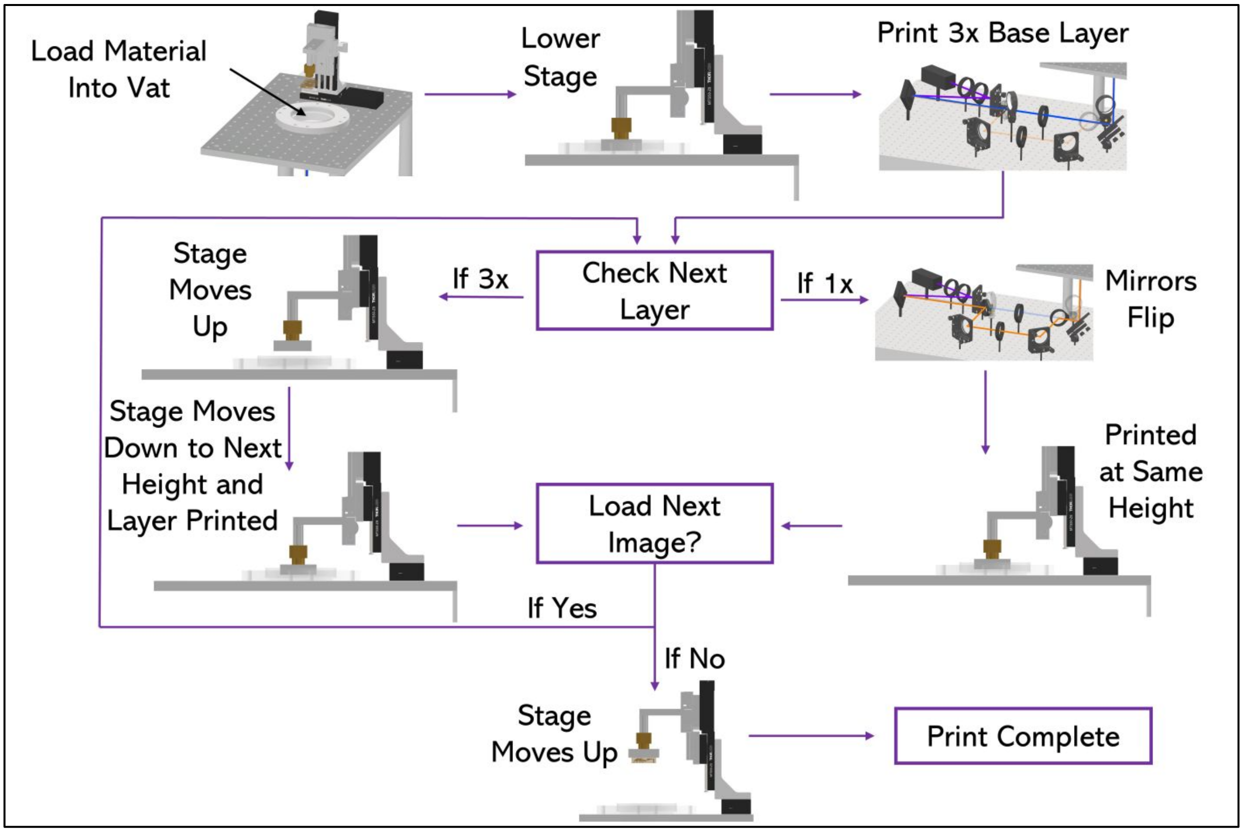

Figure S4. *MPS process overview. Printing process start to finish (top to bottom).*

To avoid any changes in image focus over time, the system's alignment is regularly checked and adjusted. To do so, the focus is checked for the 1× lens and 3× lens individually at the surface of the material vat using a camera and various pixel grid patterns. The respective lenses are mounted on linear manual stages to easily adjust the focus, without having to realign the entire system. Once a fine focus is achieved, a simple series of negative crosshair features can be printed by 3× and a set of positive crosshair features (which should align on the inside of the negative crosshair) can be printed by 1× to fine tune the lateral alignment of the two pathways. This sample is imaged, and an imaging processing algorithm built into the 3D slicer will calculate the correct X and Y image offset to correct for any final discrepancy in system alignment, applying it directly to any masks created for printing.

4. Optimization of resin formulation

Two optical properties were optimized: the chosen material should crosslink or polymerize at UV or near UV wavelength and the crosslinked material should be transparent. These optical properties can be modulated by various types and concentrations of photo-initiators, and photo-absorbers.

We first choose Poly(ethyleneglycol) diacrylate (PEGDA) of 250 molecular weight as the base material due to its excellent properties of swelling-resistance and impermeability to water. Then, two widely used photo-initiators were evaluated with ultraviolet (UV, 365 nm) and near ultraviolet (NUV, 405 nm) light sources, phenylbis(2,4,6-trimethylbenzoyl) phosphine oxide (Irgacure 819, Sigma-Aldrich) and Lithium phenyl-2,4,6-trimethylbenzoylphosphinate (LAP, synthesized in lab). Since LAP was found to be insoluble in 100% PEGDA (250 Da) resin, Irgacure 819 was chosen as the photo-initiator **(Fig. S5a)**.

Next, a variety of photo-absorbers were evaluated. With light-based printing, choosing the right photo-absorber is important to obtain the highest printing resolution. Nine photo-initiators were chosen based on their wide use in the field. These included 2-isopropylthioxanthone (ITX), Sudan-I (SI), Martius Yellow (MY), 2-nitrophenyl phenyl sulfide (NPS), 2,2,6,6-tetramethylpiperidine 1-oxyl (TEMPO), Tinuvin 234 (TIN), Orange G (OG), Quinoline Yellow (QY), and Tartrazine (Tart). The photo-absorbers were then screened based on three key requirements: solubility, spectrum matching with UV and NUV wavelengths, and optical transparency OG, QY, and Tart are found to be insoluble in PEGDA (250mw) resin **(Fig. S5a)**. TEMPO and TIN showed minimal spectral overlap with the target wavelengths of the chosen light sources, LED with peak at 365 nm and Laser with peak at 405 nm (**Fig. S5b)**. Insufficient spectral overlap will result in unwanted polymerization and prevent printing of voids and/or channels. Among the remaining photo-absorbers, ITX was found to be transparent while SI, MY and NPS showed yellow or orange colors **(Fig. S5c)**. ITX was the last man standing and was selected for this work. An ideal material formulation compatible with MPS exhibiting high transparency, water impermeability, and durability has been identified and was used in all outlined experiements. This included PEGDA (250mw) as the base material, Irgacure 819 as the photo-initiator, and ITX as the photo-absorber.

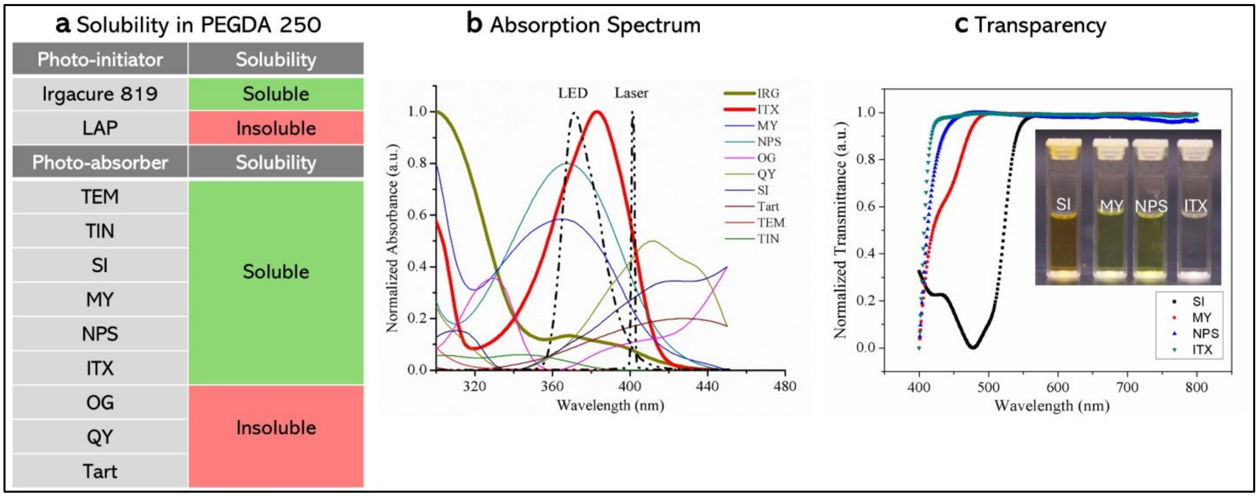

Figure S5. *Photo-absorber screening process overview. Nine photo-absorbers selected from their wide use in the field and narrowed down based on three key requirements for this work.* ***a*** *Solubility in PEGDA 250 (base material) was first evaluated, leaving six photo-absorbers.* ***b*** *The absorption spectrum of the photo-absorbers was evaluated comparing them to that of a UV LED and laser light source, leaving four absorbers.* ***c*** *The remaining photo-absorbers were tested for transparency, and ITX was identified as the best candidate, fitting all of the evaluation criteria.*

5. Computation fluid dynamics (CFD)

CFD. Computational fluid dynamics analysis was completed on the mixers to determine real world mixing efficiency.

Governing equations. The concentration field for the red and the blue dye in the domain is calculated by solving two independent convection-diffusion equations at steady-state conditions.

$$\nabla \cdot (D_r \nabla c_r) - \nabla \cdot (\vec{V} c_r) = 0. \quad (1)$$

$$\nabla \cdot (D_b \nabla c_b) - \nabla \cdot (\vec{V} c_b) = 0. \quad (2)$$

Where D_r and c_r are the diffusivity and the concentration of the red dye, and D_b and c_b are the diffusivity and the concentration of the blue dye, respectively. The independence of Eq. 1 and Eq. 2 is a valid assumption at low solute concentrations where the diffusivity of the red dye is independent of the concentration of the blue dye and vice versa. The velocity field $\vec{V}$ in Eq. 1 and Eq. 2 is computed by solving the incompressible continuity and Navier-Stokes equations at steady-state conditions.

$$\nabla . \vec{V} = 0. \quad (3)$$

$$(\vec{V} \cdot \nabla)\vec{V} = -\nabla p + \mu \nabla^2 \vec{V}. \quad (4)$$

Where p is the pressure field and μ is the dynamic viscosity of the solvent fluid. Here, we assumed that the velocity field is independent of the concentration field. This is valid when the concentration of the solute is small; thus, its effect on the density and the viscosity of the fluid is negligible.

Determining the diffusion coefficients. The diffusivity of a solute in a solvent can be estimated by the Stokes-Einstein relation.

$$D = \frac{kT}{6\pi \eta R_h}. \quad (5)$$

Where k is the Boltzmann constant, T is the temperature of the solution, η is the kinematic viscosity of the solvent, and R_h is the hydrodynamic radius of the solute molecules. The hydrodynamic radius for FITC-Dextran $M_w = 150 \times 10^3$ g/mol (blue dye) is $R_{h,b} \approx 85$ Å according to the manufacturer Sigma-Aldrich and the hydrodynamic radius for RITC-Dextran $M_w = 10 \times 10^3$ g/mol (red dye) is $R_{h,r} \approx 23.6$ Å according to the manufacturer TdB labs. These values are close to those obtained by empirical relation $R_h = 0.488 M_W{}^{0.437}$ for dextran [1]. Using Eq. 5 for $T = 20$ °C, and $\eta = 0.001 \frac{\text{kg}}{\text{m.s}}$ for water; we determined $D_b = 2.52 \times 10^{-11} \text{m}^2/\text{s}$ and, $D_r = 9.09 \times 10^{-11} \text{m}^2/\text{s}$. The calculated diffusion coefficients are consistent with the empirical relation $D = 7.69 \times 10^{-5} M_W{}^{-0.48}$ [2, 3].

Simulation setup. Water enters from each inlet at the mass flow rate $\dot{m} = 8.31 \times 10^{-9}$ kg/s and atmospheric pressure is set at the outlet. The no-slip boundary condition is imposed on all the channel walls. We defined two scalar transport equations in Fluent corresponding to Eq. 1 and Eq. 2. SIMPLE scheme was chosen to solve the pressure-velocity field [4]. The Green-Gauss node-based gradient evaluation with a second-order accuracy is used for spatial discretization of the momentum equation and scalar transport equations [5].

Case No. 1 - Fixed solid wall fins. The geometry of the domain is shown in **Fig. S6**. The maximum velocity in the channel is $V_{max} = 0.000427$ m/s, and the characteristic length of the channel is $L = 0.16$ mm; thus, the Reynolds number $Re = \frac{\rho V_m L}{\mu} = 0.06$, which is well within the laminar region.

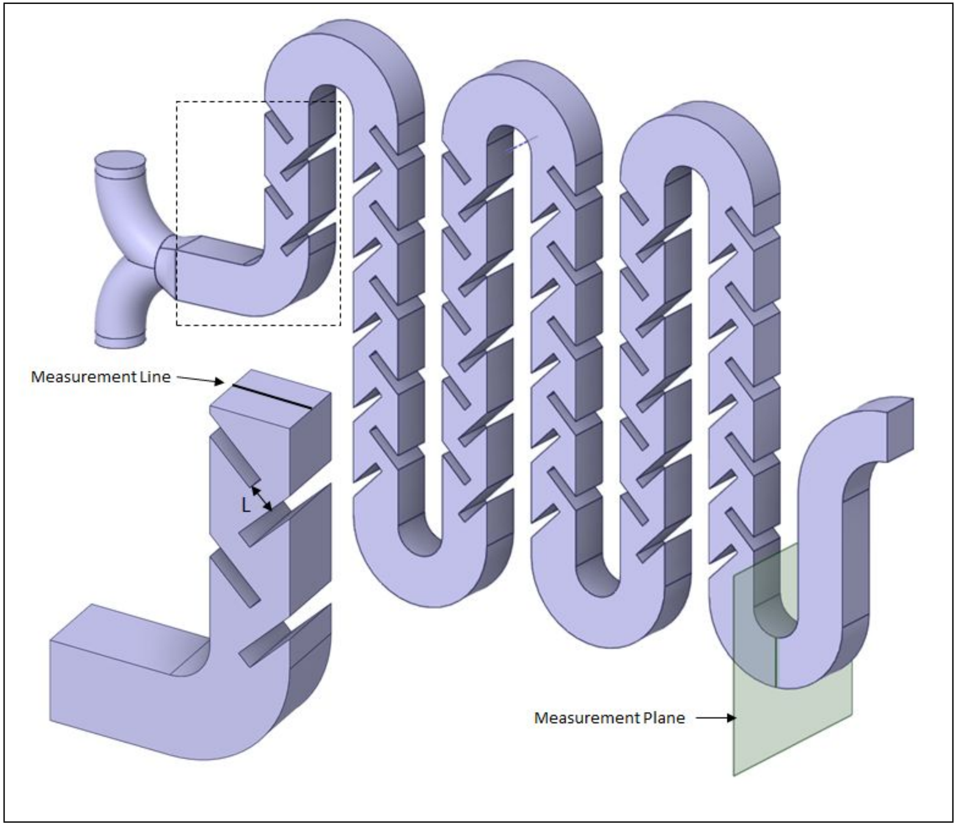

Figure S6. *Microfluidic mixer geometry number 1.*

We performed a mesh independence test to find a compromise between computation accuracy and cost. We conducted the mesh test on the section of the geometry shown in the bottom left corner of **Fig. S6**. We selected the number of mesh cells, n, per characteristic length of channel L, as the reference. We conducted five simulations for $n = 10, 15, 20, 25, 30$ which correspond to the total number of tetrahedral cells $N = 769654, 3037784, 6105443, 15226105, 21375077$. We ran the simulations until the scaled residuals for the scalar equations (corresponding to Eq. 1 and Eq. 2) converged. For Eq. 1, the residual converged at 3.6×10^{-8}, 7.5×10^{-14}, 1.1×10^{-16}, 5.2×10^{-14},1.5×10^{-15}, respectively as we increased n. For Eq. 2, the residual converged at 4.8×10^{-7}, 1.4×10^{-7}, 7.7×10^{-8}, 2.2×10^{-8}, 2.5×10^{-8}, respectively.

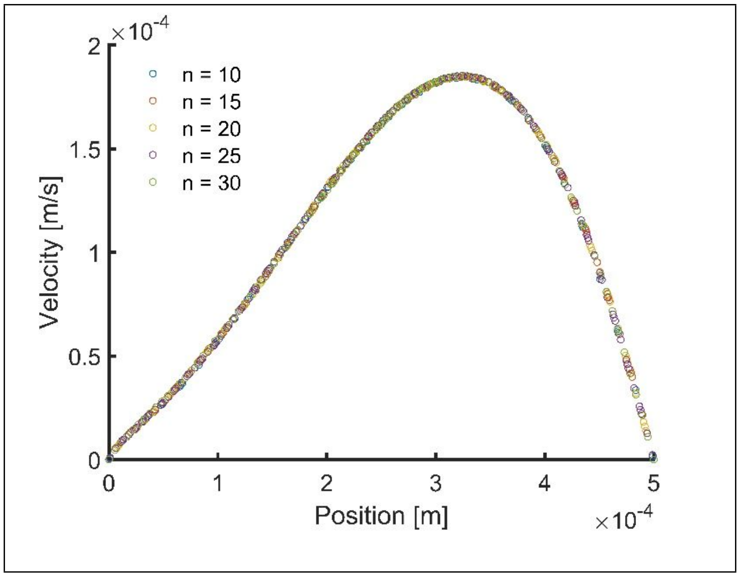

Figure S7. *Flow velocity on the centerline of the outlet.*

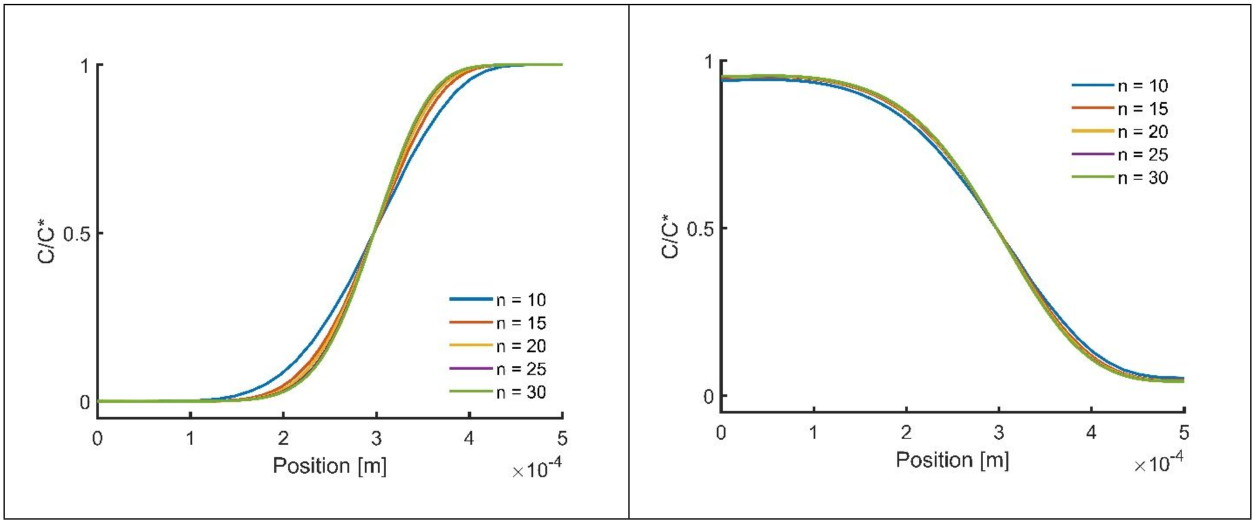

Figure S8. *Concentration of the red (left) and the blue (right) dyes on the centerline of the outlet.*

Fig. S7 shows the velocity profile on the centerline of the outlet (shown in **Fig. S6**) for the different values of n. The solution does not change with the value of n, and thus is independent from the mesh size. **Fig. S8** shows the variation in the concentration of the red and the blue dye on the centerline of the outlet. The concentration field of the red dye is still well in the mesh size-dependent region, whereas the concentration of the blue dyes is relatively less sensitive to the mesh size. As our final goal of this setup is to determine the degree of mixing between the blue and the red dye, we calculated a mixing efficiency index ME at the outlet using the results of the five simulations shown in **Fig. S9**. We will express the formula for the mixing efficiency in the next section. ME decreases from 19.48% to 18.98% when n increases from 20 to 25. This is 2.56% change, which becomes less than 1% between n = 25 and n = 30. We chose n = 20 for the primary simulation, corresponding to 172647199 mesh cells.

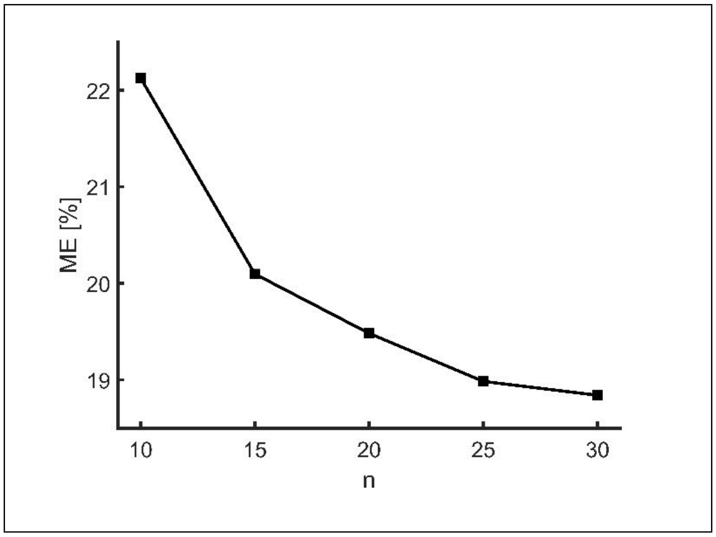

Figure S9. *Mixing efficiency (ME) versus n.*

Case No. 2 – 3D spiral fins. Fig. S10 shows the third geometry studied in this work. We performed a mesh dependency test on the entire domain using three mesh element lengths of $l = 1.9 \times 10^{-5}\ m$, $l = 1.2 \times 10^{-5}\ m$ which correspond to 8149834 and 36412425 cells, respectively. In the first trial, the residuals corresponding to equation 1 and equation 2 converged at 1.32×10^{-9} and 3.36×10^{-13}, respectively. For the second trial the residuals converged at 1.47×10^{-10} and 8.48×10^{-12}, respectively.

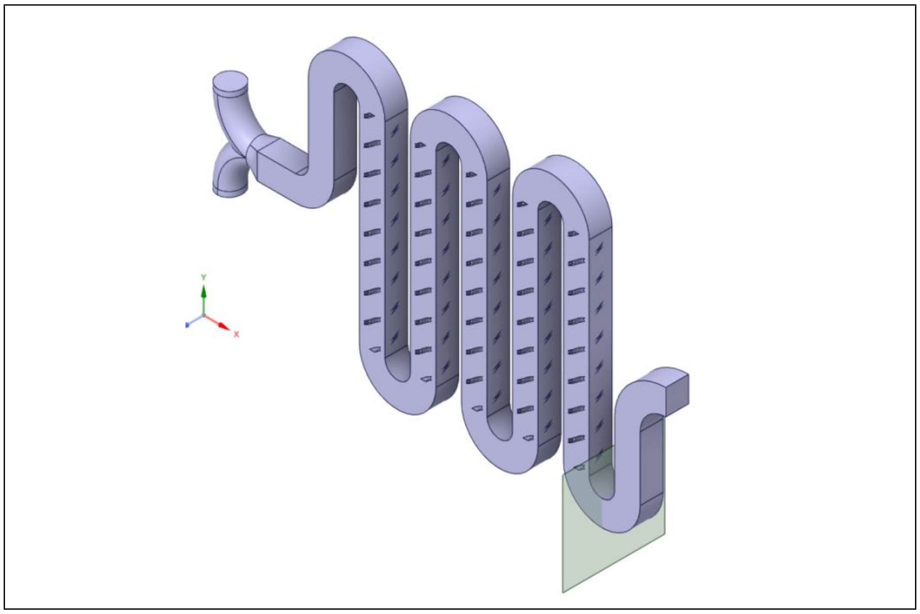

Figure S10. *Microfluidic mixer geometry number 2.*

The velocity profile on the centerline of the measurement plane is shown in **Fig. S11**. The changes in the velocity are negligible as the result of reducing the mesh size and we conclude that the velocity field is independent of the mesh size. **Fig. S12** shows the concentration of the red and blue dye on the line of interest. The concentration of the red (**Fig. S12a**) and the blue dye (**Fig. S12b**) on the centerline is sensitive to the mesh size. Although the concentration field is sensitive to the mesh size, the differences are very small. The micing efficiency changes from 99.6% to 99.18%

as the mesh size decreases. This is less than 1% change and we are satisfied with the current results and conclude that no further mesh refinement is necessary.

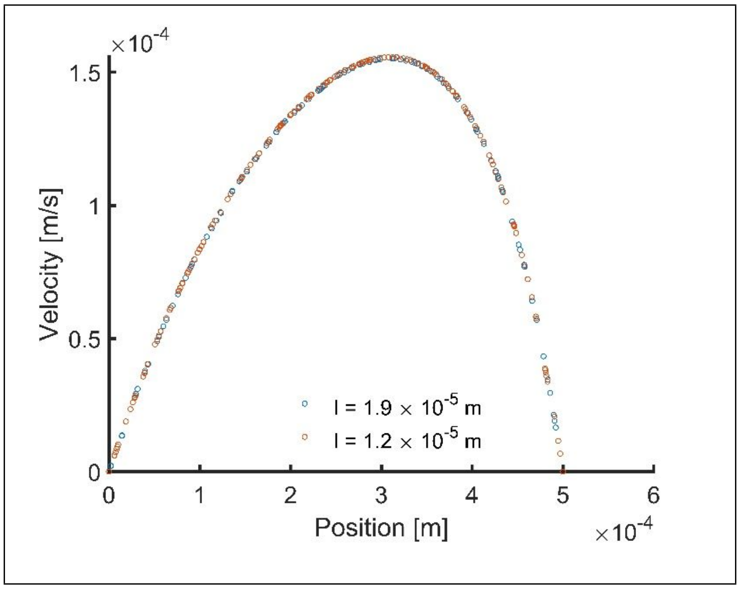

Figure S11. Flow velocity on the centerline of the measurement plane.

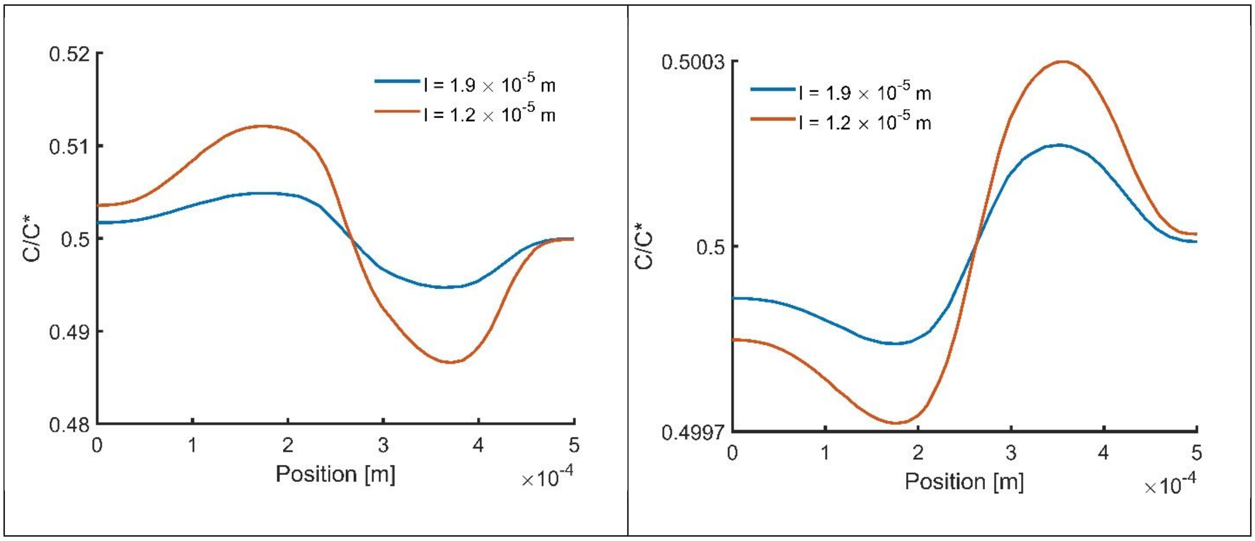

***Figure S12.** Concentration of the red (left) and the blue (right) dyes on the centerline of the outlet.*

Case No. 3 – Herringbone pattern fins. Fig. S13 shows the second geometry we studied in this work. The maximum velocity in the channel is $\mathrm{V_{max}} = 0.00019\ \mathrm{m/s}$. The characteristic length of the channel is $\mathrm{L} = 0.35\ \mathrm{mm}$ which gives $Re = \frac{\rho V_m L}{\mu} = 0.04$. We conducted the mesh dependence test on the entire domain. For the first trial, we chose the length of a mesh cell $l = 1.9 \times 10^{-5}\ m$ which corresponds to 9235333 tetrahedral cells on the entire domain. In each trial we run the simulation until the residuals corresponding to Eq. 1 and Eq. 1 converge. For the first trial the residuals converge at 1.44×10^{-9} and 6.31×10^{-8} for Eq. 1 and Eq. 2 respectively. For the second trial we set $l = 1.2 \times 10^{-5}\ m$ which results in 36433691 total mesh cells. The residuals corresponding to Eq. 1 and Eq. 2 converge at 1.62×10^{-9} and 1.42×10^{-8}, respectively. **Fig. S14** shows the velocity profile on the centerline of the measurement plane

shown in **Fig. S13**. The change in the velocity profile is negligible as we decreased the mesh size and thus, we conclude that the velocity profile is in the mesh independent region. The effect of mesh size on the concentration profile on the centerline at the measurement plane is depicted in **Fig. S15**. Finally, we calculate the $ME = 68.38\%$ and $ME = 66.2\%$ corresponding to $l = 1.9 \times 10^{-5}\ m$ and $1.2 \times 10^{-5}\ m$, respectively. This change is 3.19% and we expect it to become smaller with decreasing the mesh size and thus we conclude that the results for $1.2 \times 10^{-5}\ m$ are accurate enough for our purpose.

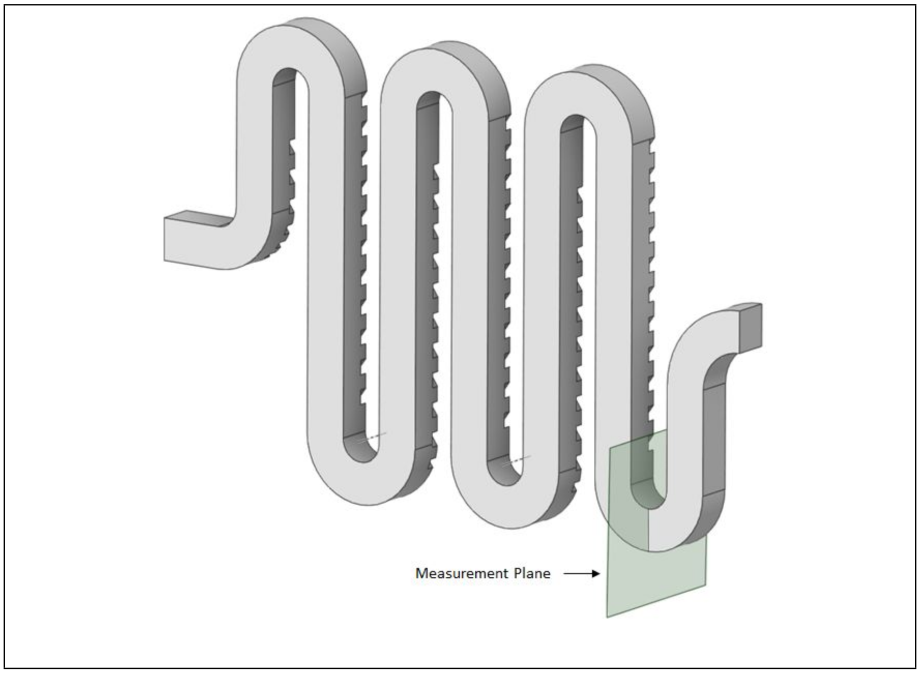

Figure S13. *Microfluidic mixer geometry number 3.*

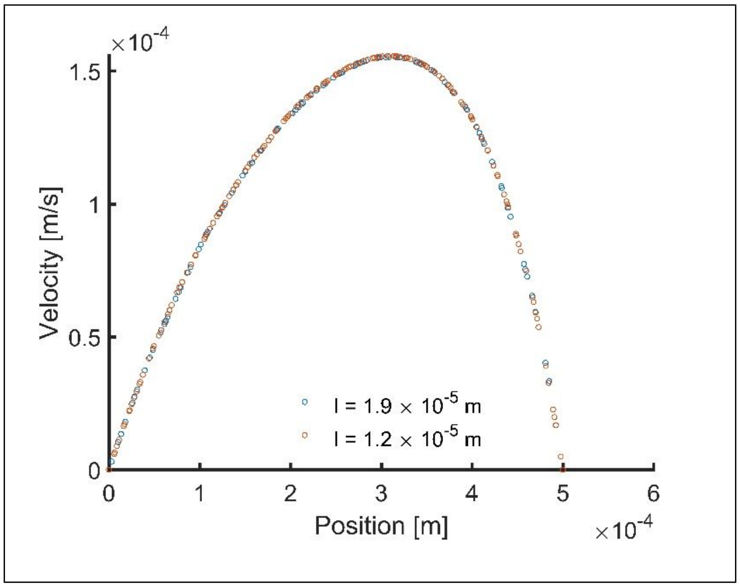

Figure S14. *Flow velocity on the centerline of the measurement plane.*

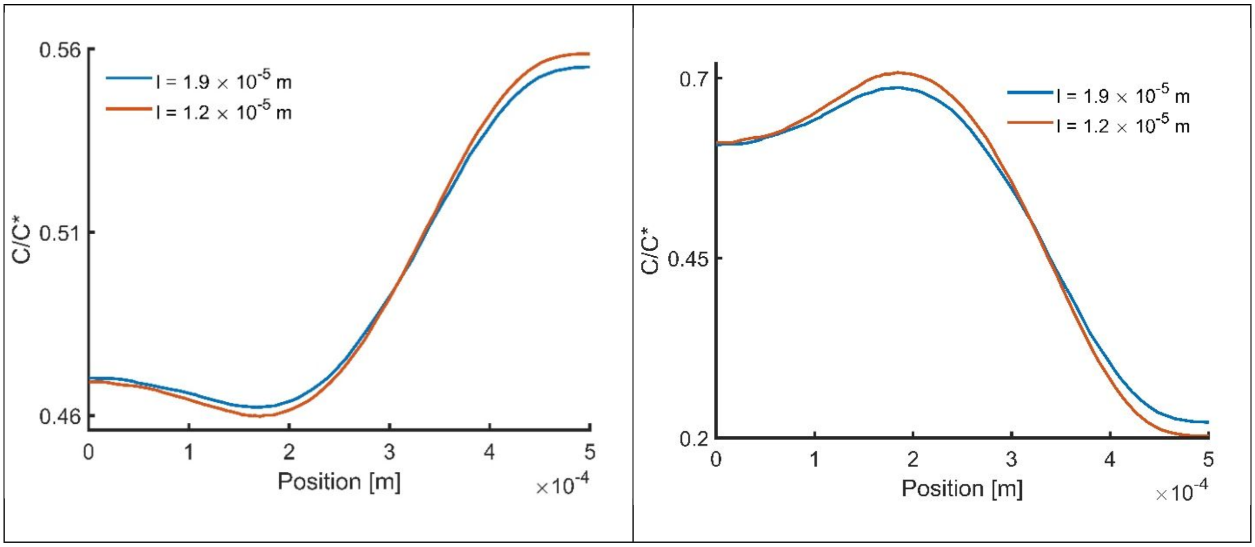

Figure S15. *Concentration of the red (left) and the blue (right) dyes on the centerline of the outlet.*

Calculation of mixing efficiency. Mixing efficiency of one species in a homogenous medium at any cross section A of a channel can be calculated by the following relation.[6, 7]

$$ME = [1 - \frac{\int |C - C_\infty| dA}{\int |C_0 - C_\infty| dA}] \times 100 \qquad (6)$$

Here we are interested in calculating the mixing efficiency of two species with each other and therefore we define a mixing ratio between the red and the blue dye as follows.

$$r = \begin{cases} C_r/C_b, & C_b > C_r \\ C_b/C_r, & C_r \geq C_b \end{cases}$$

This relation ensures that $0 \leq r \leq 1$ where $r = 0$ and $r = 1$ correspond to no mixing and well-mixed conditions at any given point in the domain. Now we can use Eq. 6 to find the average value of r for any cross section of the channel which we call the mixing efficiency between the red and the blue dye.

$$ME = [1 - \frac{\int |r - r_\infty| dA}{\int |r_0 - r_\infty| dA}] \times 100 \qquad (7)$$

$ME = 0\%$ at the inlet and it will increase downstream of the channel with a maximum value of 100 %.

6. Microfluidic mixers

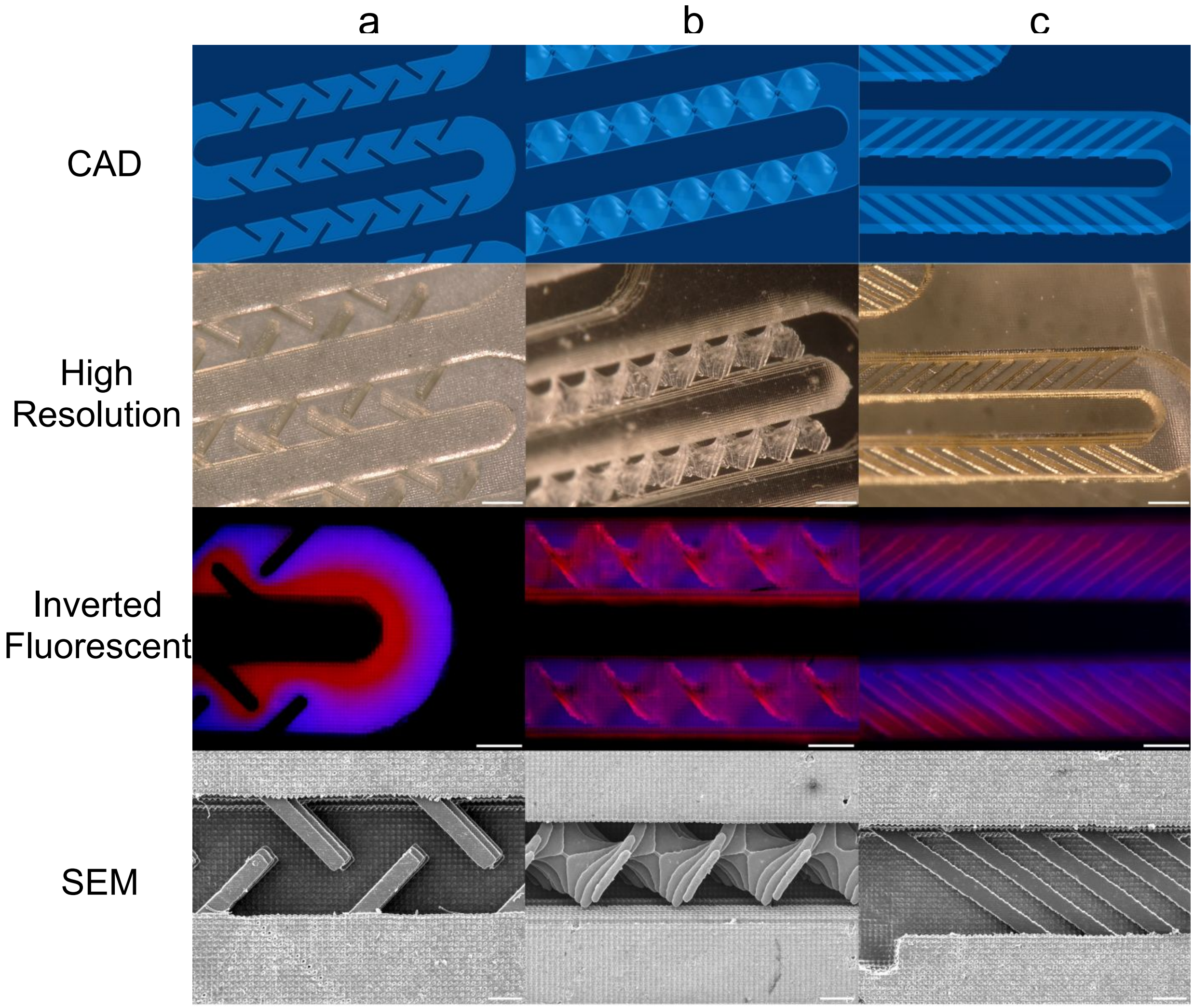

Figure S16. *Additional images for each mixer design including **a** fixed solid wall **b** 3D spiral and **c** herringbone design. Respective isometric CAD, HIROX, inverted fluorescent, and SEM images are shown. Scale bars are 500 µm (High-res), 500 µm (Fluorescent), and 200 µm (SEM).*

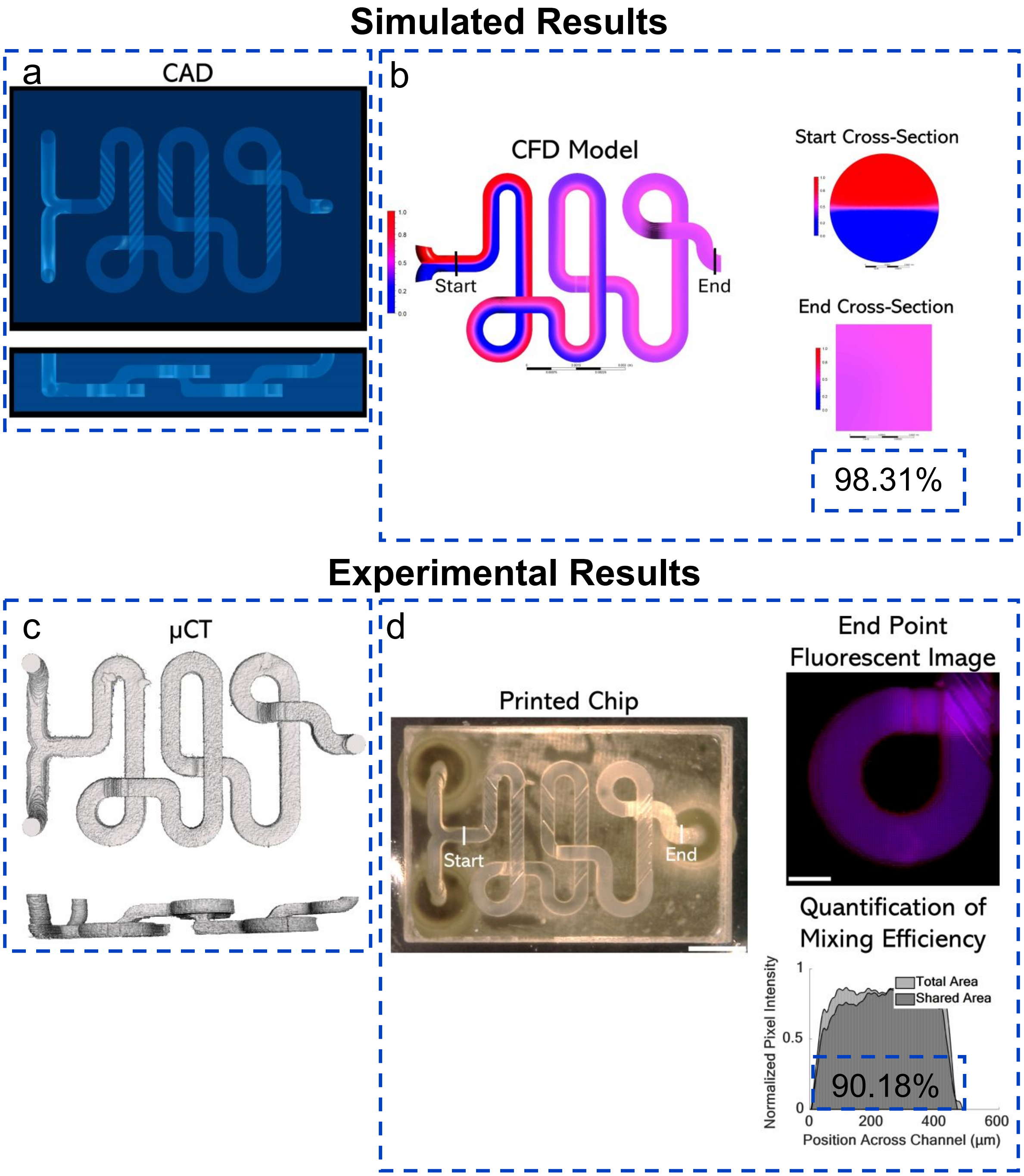

Figure S17. a *CAD of complex 3D microfluidic mixer including top and side view. Design includes herringbone pattern on bottom of channels on two planes in 3D space.* ***b*** *CFD results include top view and start/end cross section views.* ***c*** *microCT reconstruction of microfluidic mixer with same model views.* ***d*** *Printed result, top view and end point fluorescent image with mixing efficiency graph. Scale bars are 2.5 mm and 500 µm.*

CHIP DESIGN	EXPERIMENTAL RESULTS	CFD RESULTS
Fixed Walls	83.25%	83.39%
3D Fins	90.55%	99.18%
Herringbone	74.61%	66.20%
2-Plane Herringbone	90.18%	98.31%
3-Plane All Features	91.01%	93.10%

Figure S18. *Mixing efficiency summary table.*

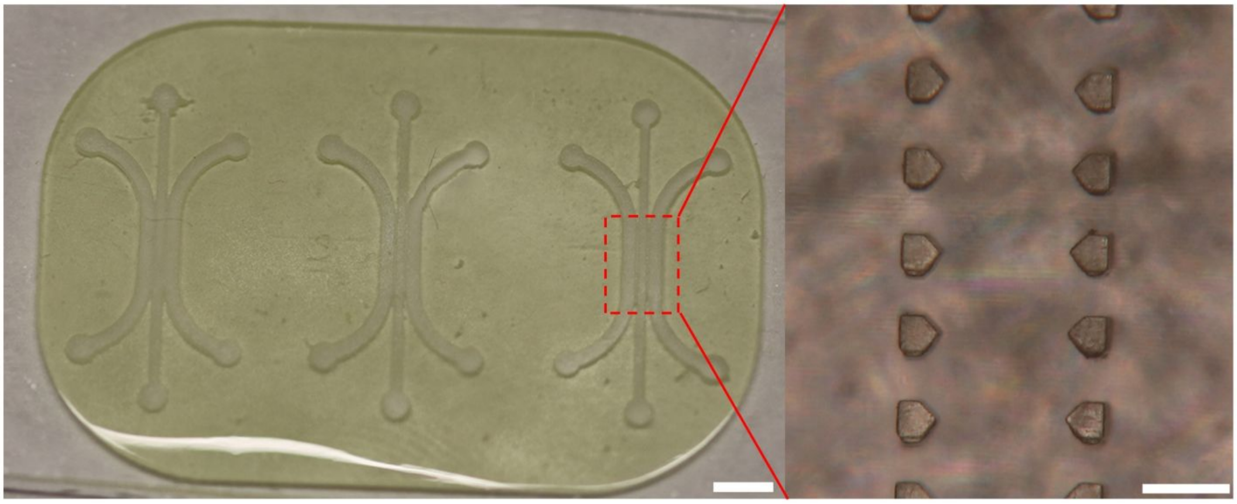

Figure S19. *Printed three-channel cell communication chip. Zoom in of 1× features inside each chip. Scale bars are 2 mm and 200 µm.*

References

1. Oliver, J. D.; Anderson, S.; Troy, J. L.; Brenner, B. M.; Deen, W. M. Determination of Glomerular Size-Selectivity in the Normal Rat with Ficoll'. *Journal of the American Society of Nephrology* **1992**, *3* (2), 214-228.

2. Berk, D. A.; Yuan, F.; Leunig, M.; Jain, R. K. Fluorescence Photobleaching with Spatial Fourier Analysis: Measurement of Diffusion in Light-Scattering Media. *Biophysical Journal* **1993**, *65* (6), 2428-2436. DOI: 10.1016/S0006-3495(93)81326-2.

3. Arrio-Dupont, M.; Cribier, S.; Foucault, G.; Devaux, P. F.; D'Albis, A. Diffusion of Fluorescently Labeled Macromolecules in Cultured Muscle Cells. *Biophysical Journal* **1996**, *70* (5), 2327-2332. DOI: 10.1016/S0006-3495(96)79798-9.

4. Patankar, S. *Numerical Heat Transfer and Fluid Flow*; 1980.

5. Holmes, D.; Connell, S. Solution of the 2d Navier-Stokes Equations on Unstructured Adaptive Grids. *9th Computational Fluid Dynamics Conference* **1989**.

6. Erickson, D.; Li, D. Influence of Surface Heterogeneity on Electrokinetically Driven Microfluidic Mixing. *Langmuir* **2002**, *18* (5), 1883–1892.

7. Jeon, N. L.; Dertinger, S. K. W.; Chiu, D. T.; Choi, I. S.; Stroock, A. D.; Whitesides, G. M. Generation of Solution and Surface Gradients Using Microfluidic Systems. *Langmuir* **2000**, *16* (22), 8311-8316.
